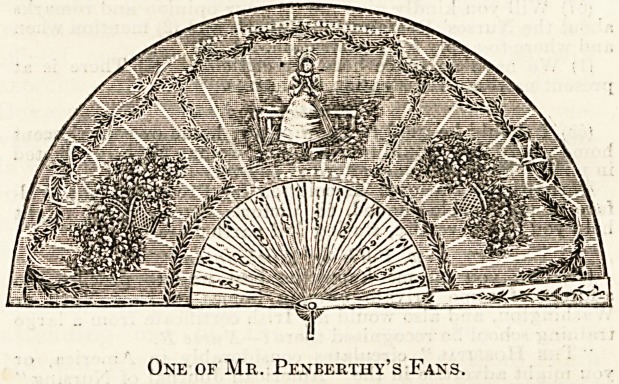# The Hospital. Nursing Section

**Published:** 1905-12-09

**Authors:** 


### The Hospital.

**Nursing Section.**

Contributions for "THE HOSPITAL," should be addressed to the EDITOR, 'THE HOSPITAL"
NURSING SECTION, 28 & 29 Southampton Street, Strand, London, W.C.

No. 1,002.—VOL. XXXIX. SATURDAY, DECEMBER 9, 1905.

### Notes on News from the Nursing World.

### OUR CHRISTMAS DISTRIBUTION.

WE remind our readers that Saturday, December 16, is the last day for sending in contributions to our Christmas Distribution, and that the articles presented will be on view at the offices of THE HOSPITAL on Tuesday afternoon, the 19th, between 3 and 5 P.M. Nurses or any contributors will be welcome. Whilst gratefully acknowledging the help we have already received, we earnestly hope that during the next few days the carriers will be kept busy in delivering parcels intended for the benefit of patients in hospitals and infirmaries. So much more numerous than usual are the appeals which reach us for participation in the distribution, that we feel compelled to press the matter upon the attention of all who are able and willing to afford assistance. The assistant matron of a large infirmary, pleading on behalf of the inmates of that institution, says, "They nearly always come in with scarcely any clothing, and that is often only fit to be destroyed." Since last week we have received parcels from Miss Young, Faringdon; Miss Elgar, Hunstanton; and Mrs. George Kinnear, Montrose. All contributions should be addressed to the Editor, 28 and 29 Southampton Street, Strand, London, W.C., and should have "Clothing Distribution" written outside.

### NURSES AND POLITICAL ORGANISATIONS

THE President of the Kensington Branch of the Women's Liberal Unionist Association writes to us to-day in defence of the meeting to which we took exception a fortnight ago. The meeting, as she herself says, was held under the auspices of the Association with the view of giving the members "real information on both sides of the important question of State Registration." This is a rather different version of the proceedings from the report furnished to the daily press, upon which our comments were founded. But we adhere to our opinion that officials of nursing bodies will do well to refuse to take part in gatherings which are got up under the auspices of an avowedly political organisation, whether Liberal, Liberal Unionist, Conservative, or Home Rule. We emphasise this point all the more in view of the prospect of an early Dissolution of Parliament, when it will be the duty of such officials to do their utmost to keep nursing topics outside the pale of party politics. If uninformed candidates for seats in the House of Commons really desire to obtain authoritative information respecting objections to State Registration, or the arguments urged in its favour, there are plenty of channels open to them without the attendance of representatives of nursing associations at meetings convened by party organisations.

### A DREAD OF NIGHT DUTY.

AN extraordinary state of affairs prevails at Battle Workhouse. There have lately been such frequent resignations of nurses that the Guardians appointed a Committee to inquire into the cause, and they ascertained that it is chiefly an intense dislike to night duty, which is greatly augmented by absence of work, their time never being fully occupied. The Committee, with the assent of the Guardians, arranged that "the nurses on night duty can retire to rest and be called should necessity arise." We sympathise with nurses who find that they have nothing particular to do in the quiet hours of the night; but this is neither a sufficient reason for dreading night duty, nor does it justify the withdrawal of nurses from the wards. Persons who, like the nurses at Battle, have "fits of nerves," are unsuited for their occupation. We think that the Guardians had better put up with further changes in the staff than leave the sick under their care without constant attendance at night.

### £10,000 FOR DISTRICT NURSING.

PERMANENT provision has been made for the maintenance of five district nurses in the Isle of Man by the gift of the magnificent sum of £10,400. This sum the trustees under the will of the late Mr. Henry Bloom Noble, who left the bulk of his large estate for charitable and benevolent purposes, principally in the island, consider could not be better applied than to the nursing of the sick poor, more especially as they know that Mr. Noble was intensely interested in all movements for the relief of suffering. Many district nursing associations in large towns would be glad to receive a legacy ensuring a sufficient income for one nurse; and it may be hoped that Mr. Noble's munificence will indirectly benefit other parts of the United Kingdom by suggesting to rich people who have no relatives in need of their money the idea of following his example.

### NURSES AND THEIR TEA.

THE Hereford Guardians have been occupied in discussing the question of the supply of tea to the workhouse officers. It appears that for thirty years the officers have had different tea from the inmates of the workhouse, and until a few months ago the tea for the officers cost 1s. 10d. per lb., and for the inmates 1s. But on September 23 the workhouse Master was instructed to issue tea from the general supply for everyone throughout the house. Then the nurses refused to drink the tea, because of its inferior quality, and bought some on their own account, with the result that on October 21 a resolution was passed allowing the officers once more a tea at a higher price than that given to the inmates. After considerable discussion a motion to take away the privilege was lost by 16 votes to 12, and the nurses at Hereford Workhouse Infirmary will therefore continue to drink the superior tea to which they have become accustomed. As nurses attach so much importance to tea, we think that the Hereford Guardians are wise in their generation.

### EVENING FROCKS IN AUSTRALIA.

A small euchre party and dance, organised by the Superintendent of the East Melbourne Nurses' Home, for the benefit of the Nurses' Sick Fund, was held in Melbourne during October, and was well attended. The view of some English matrons, that hospital sisters and nurses should not indulge in evening frocks, does not find favour at the Antipodes. The nurses in that country have not, as they put it, much time for frivolity; but they object to being deprived of the right to indulge in it—when they have the opportunity.

### ST. BARTHOLOMEW'S HOSPITAL.

The Winter Social Gathering of St. Bartholomew's League took place on Saturday in the Medical School of the hospital. There was an extra large attendance as the Provisional Committee of the National Council of Nurses of Great Britain and Ireland were the guests of the League. Miss Isla Stewart, matron of St. Bartholomew's, as President of the League, received the visitors, who were regaled with refreshments and music of a high order of merit. The nursing profession was well represented by numerous leagues and societies, as well as by the heads of many of the largest training schools, all of them old "Bart's" nurses.

### NURSES' STALL AT A BAZAAR.

At a bazaar held last month at Kingston-on-Thames in connection with St. Luke's Church, one of the features was a nurses' stall, the nurses being members of the Kingston Poor-Law Infirmary staff. The substantial sum of £12 13s. was realised by the sale of articles which were the handiwork of the stallholders.

### A MIDWIFE COMMITTED FOR MANSLAUGHTER.

At an inquest recently held in South Melbourne, medical evidence was given to show that the death of the woman was due to hæmorrhage, but that if a trained midwife had been employed her life might have been saved. The coroner eventually found that the person calling herself a midwife who attended the case had either displayed culpable ignorance or gross negligence, and committed her to trial on a charge of "manslaughter." This incident should serve as a warning to some of the midwives in the United Kingdom, who, according to evidence given before the Central Midwives Board last week, understand neither the need of washing patients, of keeping their hands clean, nor of learning how to use a thermometer.

### ACTION BY A NURSE.

An action was brought at Clerkenwell County Court last week by Miss Lucy Ridsdale, of the Nurses' Hostel, against the North Metropolitan Tramway Company, to recover damages for injuries sustained on account of alleged negligence by the company's servants. The medical evidence was to the effect that she had been suffering from a serious case of water on the knee, and she herself stated that she had been only able to do ten weeks' work between the date of the accident on January 25 until September 15, her loss being at the rate of two and a half guineas per week, with board. But several passengers by the same tram who were called as witnesses said that she left the car when it was in motion in spite of being warned not to do so; and in the face of conflicting statements, we are not surprised that the special jury were discharged because they could not agree. At any rate, the obvious moral is not to leave a tramcar under any circumstances until it has come to a standstill.

### A RESULT OF FIRST-AID LECTURES.

A course of simple nursing and first-aid lectures having been given to a large class of girls, members of the Girls' Friendly Society, in a northern country parish, the following incident shows that the efforts of the lecturer were not quite in vain, and that the hearers had derived more profit than was, at the time, apparent. The mother of one of the girls rose one morning soon after 4 A.M. intent on the performance of some domestic task; in getting out of bed she ruptured a varicose vein. Rousing her husband she sent him for her daughter, but, alas, the sight of blood was too much for the girl and she was quite powerless to put her knowledge into practical use. Seeing this, with great presence of mind, the old lady took command of the situation and proceeded to apply the second-hand lessons learnt by her daughter's repetition of what she had been taught. She ordered the girl to give her a thin cotton apron, which was hanging at the foot of the bed, then to fetch her a short stick, even indicating the spot where this would be found—*i.e.* in the yard by the pump. This done, she applied the apron as a tourniquet over the bleeding point, inserted the stick, and tightened the apron until the hæmorrhage was controlled. The doctor was now sent for, and as the good folk live on an island it was nearly 7 A.M. before he arrived. In describing the incident the old lady regrets that she forgot to elevate the limb in addition to applying the tourniquet, and ends the story by remarking in a triumphant tone: "And so you see I 'amberanced' myself."

### SHORT ITEMS.

The King has conferred the decoration of the Royal Red Cross upon Mrs. Isabel May Clay, Mrs. Violet Harriet Clay, and Miss Alice Mabel Purkis, in recognition of the services rendered by them at Dharmsala after the earthquake which occurred on April 4, 1905.—In the hope of exciting or stimulating interest in the Royal National Pension Fund for Nurses at Hull, it has been arranged for the Secretary, Mr. Louis Dick, to give an address at the Royal Infirmary on the afternoon of Thursday, December 14, at 3 P.M. Not only Queen's nurses, but all who care to attend, will be welcomed at the meeting.—The name of the sister whose death was announced last week at Southport should have been given as Rickards.

### The Nursing Outlook.

"From magnanimity, all fear above;
From nobler recompense, above applause,
Which owes to man's short outlook all its charm."

### THE EVOLUTION OF DISTRICT NURSING.

Mr. William Rathbone of Liverpool would indeed have been happy could he have lived to see the complete evolution of district nursing in this country. Looking back we remember the first attempt at district nursing was brought to our notice by a request to admit bible-women to hospital wards for six months' training in nursing. This movement commenced about 1868, and continued for a good many years on these restricted lines. To-day, although this system exists, the nurse's duties are quite distinct from those of the bible-woman, and the nurses reside in their districts working in cooperation with the local doctors, hospitals and dispensaries. They receive two years' general training in a recognised hospital, then instruction in midwifery, and then one year's training in district nursing under the direction of a superintendent sister. We thus see how Mrs. Ranyard's idea, though started tentatively, which was sound and good, has fructified and developed unto a great system like that of district nursing in the present day, which enables the sick poor to obtain in their own homes the help of a highly-trained hospital nurse, whose services render it possible for cases to be treated at home, which would otherwise have to go to a hospital.

Mr. Rathbone's great work was largely done in connection with Queen Victoria's Jubilee Institute for Nurses, which was founded in 1887. From small beginnings, and despite many serious difficulties, the Jubilee Institute has succeeded in admirably fulfiling its object, which is to train and supply nurses for the sick poor in their own homes to affiliated associations throughout the United Kingdom. At the present time some 650 such associations are affiliated, including many county associations. Each nurse is trained at some approved general hospital or infirmary, and whenever possible the Queen's nurses now hold a three years' certificate from some such institution. They are further trained in district nursing and midwifery, including the care of mothers and infants after child-birth. It is of the first importance that district nursing should have become so organised that it is now practically directed from one centre, the Queen's Jubilee Institute. The results we have just recorded show great energy and ability on the part of those responsible for the existing organisations, and we look forward to the time, when every community and city will have an ample service of district nurses, to meet all the requirements of every poor family in their own homes in the day of sickness. Already, another important feature of the work is developing rapidly—*i.e.* the after treatment of hospital cases at the patients' homes after they are discharged from the hospital when, as in operation cases, they may still require skilled attention. One district association in London attended 827 patients discharged from 22 hospitals during 1904. The standing of district nursing, too, is a high one, owing to its supervision by an efficient superintendent, whose advice in difficult cases proves most valuable.

By bringing out these facts we hope to show clearly that the decision of the Metropolitan Hospital Sunday Fund Council at its last meeting, to recommend that a sum not exceeding five per cent. of the total receipts of the Fund in each year should be set aside for distribution amongst the district nursing associations of the metropolis, was wise and prudent. The work of the Queen's nurses in London, through 22 affiliated associations, employing 145 nurses in districts which collectively cover nearly the whole of the metropolitan area, will be realised when we state, that nearly 30,000 separate patients were dealt with during 1904. The London District Nurses' Mission employs 81 nurses and attended to nearly 7,300 patients, and the North London Association employed eleven nurses with 1,500 patients during the same year. The nurses maintained at the expense of the Institute cost about £80 a year, so that the actual sum expended, on district nursing, during each twelve months by the associations just mentioned, which collectively employ 258 nurses, must exceed £20,000. Administratively the work of the district nurse should materially help the hospitals to reorganise their out-patient departments, by rendering it possible for them to absolutely restrict outpatient treatment, to those persons alone who are entitled to claim it. No one who will take the trouble to accompany one of the superintendents of a district nursing association on her rounds, and to study the work in actual being, can have any doubt, that a close co-operation between the hospitals and the district nurses must eventuate, in such a practical reform of the out-patient department, as to restrict the patients to reasonable proportions, and to effectually prevent anything in the nature of abuse. The Committee of Distribution of the Hospital Sunday Fund are at present engaged in an inquiry into the out-patient question, and we have no doubt that this point will have their full consideration.

The work of the district nurse is, however, of importance to the whole community. The more crowded and congested a district is, the more need is there for the constant presence and assistance of the district nurse. As we have pointed out more than once, disorder, discomfort, dirt and insanitation are conditions which prevail, still, to a considerable extent, in poorer households in many districts of large towns. The district nurse has to face and remove such conditions, in addition to her proper work in connection with the patient.

### Abdominal Surgery.

By Harold Burrows, M.B., F.R.C.S., Assistant Surgeon to the Bolingbroke Hospital.

### SYMPTOMS IN ABDOMINAL DISEASE.

(*Continued from Page* 117).

### Secondary Shock.

Secondary shock is produced by incessant or frequently repeated stimulation of sensory nerves. The pallor, the soft and frequent pulse, the shallow, hastened breathing, and the other symptoms by which the condition is recognised are due to a gradual exhaustion of the vital nerve centres in the brain.

Some degree of fatigue or exhaustion of these centres is almost sure to follow an abdominal operation, for the sensitive parietal peritoneum is subjected to manipulation and exposure. So the problem of how to mitigate shock is present with every case of laparotomy. The problem is to be met, also, in every case of acute peritonitis, for here the sensory nerves of the peritoneum are injured by the process of inflammation. It is worth while, therefore, in view of the importance of the subject, to consider the effects of continued or frequently repeated stimulation of sensory nerves. If the stimulation is sudden or very severe, primary shock may be induced; this has already been discussed. In other cases the sensory impressions act as stimulants to the vital centres, and at first goad them on to an abnormal activity. In consequence of this, the respiratory movements become amplified, the heart-beat becomes stronger, the body temperature rises, and the blood-pressure is increased. After a time, which varies in length according to the severity of the nerve impulses and the patient's capacity for endurance, the vital centres grow weary, and no longer are able to increase the vital functions in response to the stimulation. When this stage has been reached the breathing gradually gets less and less deep, the heart-beat loses its vigour, the blood-pressure is lowered, the body temperature falls, and the patient turns pale. He is now in the condition known as secondary shock.

### Factors which Influence the Severity of Shock.

Although the amount of shock produced is proportional to the dose of sensory stimulation which the patient receives, individuals vary a great deal as regards their response to a given dose.

As might be expected, a healthy man does not suffer so readily from secondary shock as one whose resistance has been lessened by disease; and in the former recovery from this condition is easier and more rapid than in the latter case. Some patients are more sensitive to pain than others, and they, of course, are more susceptible to shock in consequence. Neurotic people are particularly susceptible, and sometimes die from secondary shock after a comparatively small abdominal operation. In such subjects the vital centres are easily exhausted by undue stimulation. The same remark applies to patients whose nerve centres are poisoned by the products of disease, as in septic peritonitis, to those who are anæmic from constitutional causes or from recent hæmorrhage, or are intoxicated with chemical poisons, of which chloroform, perhaps, is the most important. So that, although they must not be regarded as causes of shock, the neurotic temperament, poisoning by sepsis or drugs, and anæmia are all prejudicial factors which facilitate the production of shock, and hinder recovery from this condition. In fact, any circumstance which weakens the patient will favour the occurrence of secondary shock. Fear and mental anxiety also act in this way.

A little explanation may be necessary with respect to the effect of chloroform. It might be thought, as shock is due to sensory impulses and chloroform abolishes pain, that chloroform anæsthesia would delay the onset of shock. This is not so. Although the patient when anæsthetised is unconscious of pain, yet chloroform does not prevent the passage of sensory impulses to the vital centres. Now the vital centres are not anæsthetised. If they were, they would cease to exercise their functions, and respiration and circulation would stop and the patient would die. But although not anæsthetised, they are to some extent poisoned by the chloroform, and this poisoning renders them more easily exhausted than before.

It has been shown by experiment that shock is much more readily induced when the patient and his surroundings are cold than when proper warmth is provided. A lowered temperature must be added, therefore, to the list of factors favourable to the production of shock.

### Treatment of Shock.

Although, as a rule, shock cannot be prevented, yet much may be done to check its severity. Especially is this so in the case of abdominal operations when there is time to take precautionary measures. As is natural, exhaustion of the vital nerve-centres, which is the immediate cause of the symptoms of shock, is less easily induced in those who have bodily health and mental stamina than in the feeble and neurotic. On this account it is good practice in those cases where an operation, though necessary, can be delayed, to prepare the patient for some weeks beforehand, to get his nerve-centres into training. For this purpose a period of mental repose, accompanied by moderate physical exertion in the country, is beneficial, especially if the patient be an overwrought mental worker or a neurotic society lady. As mental repose is not possible to everyone who has the ordeal of an operation in front of him, it is sometimes well to defer a final opinion concerning the necessity for operation until the time for its performance is near at hand. This hygienic preparation is the ideal practice, and the opportunity for acting in strict accordance with it is uncommon. But it is worth mentioning. Some preparation of the patient's mind may be made in nearly every case, and may be carried out more easily and effectually by the nurse than anyone else. Thus the nurse should try to render the patient confident in the outcome of the operation, doing all

ABDOMINAL SURGERY—*continued.*

she can in a quiet and unostentatious way to secure her charge from being harassed by fear and anxiety. The patient who lies awake the greater part of the night previous to an operation, through dread of what is before him, will not be in the best trim to withstand the trials which the procedure brings. The necessities of proper nursing and preparation should always be arranged and performed with this fact well in view.

(*To be continued.*)

### The Nurses' Clinic.

### THE LYING-IN PERIOD.

What is a luxury to one generation is a necessity to the next, and in the same way the attention bestowed on midwifery cases to-day would have amazed our grandmothers. The poorest mother now receives more knowledgeable care and attention than queens of bygone days. The thoughts of a middle-class mother generally turn to the provision of a trained nurse for her confinement as naturally as towards the making of the layette, and from the treatment and advice she receives from her nurse at that time much good may result. It might have been thought entirely unnecessary to remind nurses never to show their patient illustrated midwifery books, or to relate their experiences of difficult labour, if one had not recently heard of a doctor transgressing in that respect. He was, however, a Portuguese, and the incident took place in Lisbon, *autre pays autres mœurs*, perhaps our foreign sisters are not easily frightened. Never, also, be "drawn" by the patient or her friends; answer all queries courteously but guardedly, remembering how very dangerous is a little knowledge, and, if pressed, suggest that the matter should be referred to the doctor. Many a young mother has never heard of instrumental labours; then let her remain in ignorance. Should it be necessary to use forceps in her case, it is the doctor's province to inform her, and he will do so in such a manner as to rather encourage than frighten her, and as an anæsthetic will be administered she will have but a very hazy idea of what is going on.

In a private house the nurse is usually only required to act as "monthly" under the doctor's instructions, and to see that everything is prepared in the lying-in room, which her experience has shown her to be necessary. The patient is always ready to be guided by the nurse as to what to get, and she could not do better than buy one of the "accouchement outfits" sold at one guinea or 10s. 6d. A douching apparatus, Higginson's syringe, and bed-pan she should also supply, and will doubtless be glad to keep by her for other occasions.

The nurse is usually provided with a catheter and antiseptic tabloids. One word of caution about sublimate corrosive tabloids. It is safer to make the lotion some time before it will be needed, and have it thoroughly strained, as cases have been known, when a solution was required in a hurry for an intra-uterine douche, the tabloid was only partially dissolved, and solid particles were carried up and left in the uterus, setting up serious mischief. A bottle of boracic lotion will be wanted, and have some ready in a small dish to wipe the baby's eyes as soon as the head is born.

The flannel receiver is a detail often forgotten. A shawl or cot blanket answers the purpose very well, but do not omit, before wrapping up the child and laying it temporarily aside, to wipe the hands clean and dry, so that he may not carry infection to his eyes. He must be placed in a warm spot near the fire, for it may be some time before you can attend to him, and infant vitality is low.

If the baby is strong he may be bathed at once, and every day after; but if very feeble or premature, the shock of immersion is too great, and he should simply be rubbed with oil and wrapped in cotton wool to obviate the fatigue of dressing.

Supposing it is a normal, healthy infant, let it be put to the breast for a few minutes as soon as he is washed and dressed—then maybe only a drop or so—but the action of suckling has a contracting influence on the uterus, and so is beneficial to the mother. Her milk does not flow freely till the third day, when it is sometimes ushered in with a rise of temperature and a sense of fulness and burning in the breasts. Each time the child is fed the nipple should be wiped over with boracic lotion (this is a point the mother can attend to herself after the first few days), and the baby's mouth must also be attended to, for even breast-fed infants may develop thrush unless kept scrupulously clean. Should there have been any tearing of the perineum, as is sometimes the case with primiparous women, the nurse must see that the stitches are kept as aseptic as possible, bathing the parts with lotion every time urine is passed. The bowels are usually relieved by a simple enema, and all straining avoided. Some surgeons, however, prefer an aperient, and will give orders accordingly.

Douches are given chiefly according to the case, not as a routine measure. Many doctors prefer to omit them altogether, especially after a normal delivery. "Let well alone" is a sound motto.

On the third day it is customary to give an aperient, liquorice powder, senna or cascara, are all good, and may be followed by a seidlitz powder in the morning; but the mother's wishes can be considered and carried out on this point.

The diet of the mother, if left to the nurse, should be simple, abundant, and nourishing, plenty of milk, chocolate, and good soup. Alcohol is not necessary unless ordered by the doctor. Some anæmic women seem to derive benefit from stout, but the tendency is to avoid stimulants except under certain conditions.

The period of rest in bed varies, but it is a safe rule never to get up before the eleventh day, and in private houses usually not before the fourteenth. There is nothing to gain by getting up early, and often a good deal to lose. Some women think it a positive disgrace to lie in beyond the tenth day, and quote our late Queen Victoria as their example; but in these strenuous days of hurry and unrest a few days more in bed will benefit them more than slavishly following any model or tradition.

### To Nurses.

We invite contributions from any of our readers, and shall be glad to pay for "Notes on News from the Nursing World," "Incidents in a Nurse's Life," or for articles describing nursing experiences at home or abroad dealing with any nursing question from an original point of view, according to length. The minimum payment is 5s. Contributions on topical subjects are specially welcome. Notices of appointments, letters, entertainments, presentations, and deaths are not paid for, but we are always glad to receive them. All rejected manuscripts are returned in due course, and all payments for manuscripts used are made as early as possible after the beginning of each quarter.

### Christmas in the Mental Hospitals.

This year, by the kindness of representatives of well-known public and private institutions, we are able to show how Christmas is spent in the great mental hospitals. Of course, the contributions describe, and the pictures illustrate, the incidents of last Christmas, but they also furnish a fair idea of the manner in which the festival will be observed this year, with the noteworthy difference that it falls on Monday, instead of Sunday, as in 1904. There are, obviously, two points in which the patients in mental hospitals do not resemble those in general hospitals, poor-law infirmaries, or isolation hospitals, whose mode of spending Christmas has been dealt with on previous occasions in our columns. Many are in the enjoyment of excellent bodily health; all are suffering, more or less, from some form of mental disease. The Christmas entertainments therefore vary materially, and the patients themselves are able to afford considerable assistance. We freely admit that an asylum may not be the exact place one would choose in which to spend a happy Christmas, but the impression that people who have the terrible misfortune to be mentally afflicted cannot possibly enjoy themselves at that festive season of the year will be effectually corrected by the accounts furnished by our corespondents from all parts of the United Kingdom. One fact is brought out strongly—namely, that no form of entertainment appeals so much to the sufferers as a fancy dress ball. The brightness, the colour, the motion, seem to be immensely appreciated, and we assume that no ill results follow the reaction, or dancing would cease to be a feature at Christmas. The performance of plays and concerts is scarcely less popular. It is pleasant to learn how eagerly the celebration of the Christmas festival is awaited; how thoroughly the sacred, as well as the secular functions appear to be appreciated; and how entirely the members of the staff, medical superintendents and assistants, matrons and nurses of both sexes, surrender themselves to the task of brightening lives which, to a large extent, are veiled in impenetratable darkness. Pitiful as the lot of patients in mental asylums, public or private, must ever be, it is a matter for congratulation that these institutions, like general hospitals and Poor Law infirmaries, have not only moved with the times both as to equipment and nursing, but are now so often redolent of the humanising element which at one period was so frequently wanting. The methods of observing Christmas, the personal devotion which is manifested, the kindly interest which is displayed, the thoughtfulness, patience, and discretion which the occasion calls into action, bear emphatic witness to the growth of progress, and of that spirit which Christmas, of all seasons, ought to foster and extend.

### Claybury Asylum.

In hospitals and asylums signs of the Christmas festival show themselves long before the time fixed for it in the calendar, and at Claybury Asylum patients, nurses, and attendants have begun to talk of and to prepare their decorations even several weeks beforehand—busy fingers commencing to make paper chrysanthemums, lilies, roses, and carnations, and other suitable emblems of the great festival.

The healthy spirit of rivalry which exists outside our asylums is also to be met within them, and this rivalry encourages ingenious designs among the many workers of our busy hive. Those patients who hitherto appeared

Dining-room at Claybury Asylum.

**CLAYBURY ASYLUM**—*continued.*

absorbed in their own affairs, at this season of the year seem to be impelled to join with others to paint pictures, work mottoes, construct festoons, arrange snow scenes, or to invent grotesque or genial figures of Father Christmas and Santa Claus—all to celebrate the happy season. There is analogous to the "vernal impulse" a Yule-tide tendency, and the climax of this is to be seen in the numerous proverbs and maxims in Latin, English, and Welsh—for there are in Claybury numerous units of the Celtic fringe—which are made out of evergreens, holly, and laurel leaves. Clumps of mistletoe are also seen in every ward, and coloured paper is deftly used for mottoes bordered, as these are, by flowers emblematic of all the various seasons. The general effect left upon the mind is bright and cheering, and the combined influence of all these varied additions is to emphasise contentment, sympathy, and homeliness.

Kitchen at Claybury Asylum, with Male Cooks.

The musical members of the staff have also their contributions. Those who sing and play usually present, a day or two before Christmas, a comic opera under the leadership of the organist and choirmaster, and the greatest pleasure is thus given to a large audience in the great hall. If possible, a full-dress rehearsal of this takes place for the benefit of the staff on the evening previous to its production.

Christmas Day, more especially if the festival fall on a Sunday, begins with a celebration of the Holy Communion in the Asylum Chapel which is beautifully decorated by the matron and her staff encouraged by the Chaplain. The decorations are of holly, ivy, laurels, and flowers from the asylum greenhouses. This is followed by Matins and a sermon at 10.45, both services being well attended by the patients and staff. At mid-day the patients sit down to a substantial dinner of roast beef and vegetables followed by Christmas plum-pudding and dessert—apples, oranges, raisins, figs, and nuts.

The staff afterwards partake of their Christmas fare, and the extra cost of these dinners for patients and staff is about £153. Over 7,000 oranges are generally provided, £20 being spent upon dessert fruit, whilst nearly sixty pounds' weight of tobacco is issued to the patients as an "extra smoke" on that day. Over 1,600 eggs are used to make the Christmas puddings, and more than 600 lbs. of currants, 400 lbs. of raisins, 300 lbs. of sultanas, and great quantities of flour, spices, and sugar. The quantities of food material in addition to the ordinary dietary which are employed in a great asylum show the magnitude of catering for the numerous colony therein housed.

The reflection occurs that great technical knowledge and administrative skill are necessary to break the monotony of routine in a great asylum, and the various entertainments and the dances are absolutely necessary. The sports and games for men are always to the fore in these places, but forms of recreation in which all can partake are none too frequent and their appreciation is only too evident as may be seen when the expectant faces are watched at some of the weekly entertainments.

On Christmas Day in the afternoon a party of carol singers including the medical officers, choir and organist, visit the wards. The carol singing, repeated year by year, has become one of the most highly appreciated items of the day's programme, especially in the wards where the infirm and the sick remain—*i.e.* those who are unable to attend the chapel services. Passing from ward to ward with mirth which "shall not overstep the bounds of reverence," the singers tell out in tuneful and familiar strains "the old, old story of the first Christmas" and many happy memories of days long past and reminiscences of former associations are kindled by these joyful tunes which are truly songs of joy and exultation. The following is a list of the carols which were sung during the afternoon and evening last year: "Good King Wenceslas"; "Good Christian men, rejoice"; "Come, ye lofty"; "God rest you merry gentlemen"; "The First Nowell"; "Sleep, Holy Babe"; "Hymn for Christmas Day," etc.

It is significant that in this great asylum with over 2,400 inmates and over 400 staff of day and night workers, no alcoholic liquor is served, such, indeed not being necessary, nor is it asked for. Moreover the presence of alcohol as a beverage would be to many a reminder of their sad fall; and its absence on such an occasion proves that happiness can be attained to the full—testified to by appreciative expressions of contentment on all sides—without having recourse to indulgence in intoxicants.

### Leavesden Asylum.

It is, outwardly at least, an ideal Christmas when a thick hoar frost descends upon everything and the gardens present a sparkling, fairy-like scene, with the treetops looming out of a blue haze, which just manages to obscure the sun. Alas, however, the weather is not always equally pleasant; but rain or hail, snow or fog, Christmas Eve is ushered in with music and song. Last year into ward after ward of the asylum came a party of kind-hearted and merry boys and girls, accompanied by some of their elders, the girls attired *à la* Red Riding Hood and the boys in scarlet cassocks and white surplices, who sang the old, old Christmas carols, recalling pleasant memories to all. Later a neighbouring village choir serenaded us with more carols, and still later our own carollers, marshalled by our indefatigable and musical chaplain, again sang old favourites for our delectation.

Last Christmas Day, the festival falling on Sunday, there was an early Communion service for the staff at 7.30 A.M., whilst the Christmas morning service at 11 A.M. was followed by a Communion service for the patients, of whom fifty-one participated in the most reverent manner, together with some of the officials. The chapel is always beautifully and tastefully decorated with greenery and flowers by ladies of the staff, and the well-attended service is made bright by the hearty and harmonious singing of the choir. After breakfast the asylum is visited by the

St. Pancras School Band, which discourses Christmas music in the corridors, filling the whole building with sweet sounds. An excellent dinner of roast beef and plum-pudding, reflecting great credit upon the cook and all concerned, is served to all able to take it, but the sick, bed-ridden, and very old are present at Leavesden in such large numbers that many may not enjoy such luxuries because of the risk of direful consequences. Following dinner sweets, fruit, tobacco, and pipes are handed round, and a pleasant though quiet afternoon succeeds, whilst in the evening the recreation hall is filled for a lantern service, when the chaplain exhibits on the screen pictures suitable for the occasion. And so to bed and to sweet memories.

On Bank Holidays many of the patients look forward to visits from relatives and friends, who may be encountered scattered through the eighteen infirmary wards, or forming little groups dotted over the extensive floor of the recreation hall, the centre being a patient.

A few days after Christmas the patients' Christmas dance, with an interval for refreshments, takes place. Recent years have seen the number of grey heads and grey beards increase among us, so that there are many more onlookers than dancers. The Christmas dance must, notwithstanding, always take place—even if officials should have to dance together—in order to give what is undoubted pleasure to the onlookers, and to make them renew their youth by watching others trip the boards "upon the light fantastic toe." The function of Leavesden Asylum is to nurse and care for a large and hopeless population in a state of mental and bodily wreckage, agedness and feebleness being marked characteristics. Thus there cannot be quite the same active recreations for the patients as at other asylums.

The necessity of providing due recreation and entertainment for the nursing staff to counteract the effects of their discouraging, depressing, and often disagreeable work is acknowledged by all, and after the patients' dance comes the Christmas staff dance. Festoons of green leaves and paper flowers hang from the roof of the recreation hall, the stage, effectively lit with Chinese lanterns, makes an admirable

Dining-room at Leavesden Asylum.

sitting-out room, whilst the floor is as smooth and polished as dancing feet could desire. A bright-coloured throng in fancy and evening dress trip gaily along to the music of our own band, esconced behind a screen of palms. At midnight, and after refreshments are served, we all join hands and sing together "Auld Lang Syne."

### Royal Asylum, Perth.

It is many years since the festivities for Christmas and New Year became a fixed routine at James Murray's Royal Asylum, Perth. The most suitable arrangements were naturally gradually developed by experience, but now these entertainments vary only in dates. As a rule it is found desirable to fill up Christmas Day as fully as possible, beginning

Ladies' Hospital at Royal Asylum, Perth, with special service in the chapel; thereafter all patients possible dine with the officers, dinner concluding with a speech or two, there is then an adjournment to the billard-room, and a drawing-room party in the evening. The Christmas ball is generally held on the 23rd, when a number of visitors more or less intimately connected with the institution are present. Last year it was the first dance in the new ball-room, so that the attendance was unusually large and enthusiastic. With regard to the staff dance it is almost invariably held on the last night of the year, on which night no leaves of absence are granted. The staff invite their friends and make merry with them within the hospital. This arrangement when first instituted some twenty-five years ago, was most unpopular, as the old Scottish custom called "first footing" (with whisky) was in high favour. The result was disastrous to some, and, therefore, the staff ball was inaugurated. The staff at Perth numbers about ninety all told, which is a large proportion to the total of patients (145). This is accounted for by the number of separate houses, the employment of ladies as nurses, with ward-maids to do cleaning, and the maintaining of an artisan staff instead of contractors and casual labourers. Principally, however, this large aggregate is found needful, because modern asylum nursing requires a much greater number by day and by night than was formerly deemed necessary. At present there are twenty-three attendants and twenty-four nurses, solely engaged in the care of the patients. On the night of the ball there are often as many as 140 present.

Early in January the Matron of Kincarrathie entertains a large number of the ladies and gentlemen at dinner, and in the asylum kitchen the Artisans' Annual Dinner is usually very popular.

Last of all, the Christmas-tree party is given somewhere about Twelfth Night in the afternoon. This hardy annual is perhaps worthy of special note, as the ladies and gentlemen here take a very great interest in it, and entertain the children of the staff, who at present number forty-one. The children are presented with useful gifts and toys, on the careful selection of the Matron.

### West Riding Asylum, Wakefield.

Rarely if ever does the spacious entertainment hall of the West Riding Asylum appear to greater advantage than on the occasion of the patients' annual fancy dress ball. The hall lends itself readily to decorative effects; its panelled walls, its lofty windows, and orchestral gallery are always charmingly draped, harmony of colour and symmetry of design being admirably secured, whilst gay-coloured banners float down from the open-raftered roof. The fixed stage, with its handsome proscenium, provides a delightful reception-room for the house party, transformed by the magic hand of the scenic artist into a sylvan scene, with distant vistas of mountain, stream, and lake. Ferns spread their rich fronds from hanging baskets overhead, and form an aerial forest; foliage plants from the asylum gardens stand out at every point of advantage in great profusion; groups of arum lilies extend their snow-white chalices up the proscenium arch; whilst the electrician's handicraft is in evidence in the brilliant designs of electric lights along the orchestral front.

A fancy dress ball more than any other form of entertainment appeals to the patients' sense of healthy enjoyment. If anything could divert the morbid mind from its fatal self-engrossment surely the brilliant costumes of a fancy dress display, the kaleidoscopic play of colour and symmetry of figure, would do so, not to speak of the rousing strains of the asylum stringed band, which for so many years has upheld its reputation for excellence. On either side of the hall some 800 patients arrange themselves in joyous expectancy, dressed in pleasing, quaint, or fantastic costumes, with a strong vanguard of nurses in Japanese attire. At the appointed moment the guests of the evening arrive in brilliant and varied array, and, passing through a guard of sturdy helmetted firemen—the asylum brigade—headed by six stalwart Masters of Ceremonies in Claude Duval attire, they gaily make their way through the body of the hall to the stage drawing-room. The next moment, with the lively strains of the band to assist, the room becomes a labyrinth of ever-changing colour and rhythmic grace of movement, and one feels dazzled by the endless change of effect. Lancers, waltz, galop, polka,

Fancy Dress Ball at the West Riding Asylum.

quadrille, and homely march succeed each other in rapid sequence, forming a continuous crescendo of enjoyable excitement. Threading the giddy maze, patients, guests, and officials interblend in one harmonious whole, and for one evening at least it might with confidence be affirmed that no gloom haunts the minds of the inmates.

Later on comes the nurses' dance. Last year all the nurses were arrayed in exquisite Japanese costumes—a harmony in violet and yellow; they danced the "Veleta Waltz" with charming effect, the two most graceful dancers being presented with a prize by the medical director's wife amidst rounds of applause.

After refreshments the programme is proceeded with, on into the small hours of the morning, with well sustained verve. At last the grand old anthem warns all that the great event of the year has come to a close; that complete success has crowned the efforts of the many hundreds who have co-operated with single aim for the general good; that a brilliant spectacle, involving innumerable phases for the mind to dwell upon for several months to come, has been enjoyed by all alike; and that the chain of sympathy which extends from the afflicted community inside the walls to the world of more fortunate beings without has been substantially strengthened in every link. This reflection alone is a powerful factor.

A week after the patients' fancy dress ball the nursing staff have their own "Officers' Ball," at which no patients are present, and when even the official insignia of uniform is dispensed with, and each has the privilege of an invited guest; whilst the married staff extend the hospitality of the asylum to their life's partner at a most pleasurable dance. All who are familiar with the mentally afflicted recognise the strange inversion of healthy mental functions, the self-centred contemplation, the apathy of response, and especially the decline of imaginative vigour. The endeavour in the asylum is to meet this latter defect by several forms of associated entertainments. In the childhood of life one is largely fed on an aliment of fairy tales and stories of peril and adventure, and it is found that this is an aid in the formation of a faculty which is in full tide of development. But in the reductions of insanity it is necessary to step in with spectacular effects, and supply what the weakened mind fails to conjure up or realise; to give the patients, so to speak, their fairy tales as seeming objective realities, and to strive to evolve healthy emotional states which may eliminate the prevailing gloom.

The anticipatory stage of Christmas is fraught with much good; for weeks prior to the event the wards and workrooms are the scene of busy brains and lissome fingers, a veritable human beehive, and a joyous outlook dispels dark melancholy. In fact, it is in the anticipation and retrospect that so much good is secured; this is a moral agency of lasting nature. Healthy rivalry, keen competition, hearty generous co-operation are the watchwords of all striving to produce the desired effect. Nor, perhaps, is it of little moment in these days of public audits and burdened ratepayers to indicate at how trifling a cost all this pleasure and good is obtained. A careful organiser with artistic taste and judgment can readily secure most pleasing effects at very little outlay, whilst the invited guests, whose presence heightens the interest of the inmates, co-operate most powerfully, by their brilliant and more costly costumes, to enhance the spectacular effect.

"Do you not dread this excitement for your inmates?" was the question put by one. "No more than you dread the stimulating effects of a mountain climb," was the reply, "or the flushing of your lungs with the cool bracing moorland air." For in normal life the mental tides have their ebb and flow, their neap and their flood levels; but, in the large majority of the insane, a dead monotonous level obtains, and the desire at the West Riding Asylum is to correct this state of things, to flush with the emotional tides the channels of stagnant thought, stimulate healthy desires, re-vivify dormant imagination, and brace up the limp and irresolute will.

### Woodilee Asylum, Glasgow.

Christmas Day itself is not celebrated at Woodilee with any special rejoicings for the patients, because in Scotland amongst the class for whom the asylum is intended it is customary to celebrate the 1st of January rather than the 25th of December. Therefore upon New Year's Day there is an extra dietary for all, consisting of roast beef and plum-pudding, a tea with many good things in the evening, and it is followed by an entertainment consisting of dancing, interspersed with songs, recitations, etc. These festivities last from seven o'clock to ten. All the working patients are given a holiday, but, of necessity, the same pleasure cannot be given to the staff the same day, as they are required to devote the whole of their time to the patients, so a staff dance is given later on, to which every nurse and attendant is allowed the privilege of inviting one friend. A special train comes out from Glasgow, and as the asylum orchestra consists of attendants and staff, an extra orchestra is engaged for the occasion.

One entertainment is reserved specially for the children—those little ones, mostly idiots and imbeciles, who occupy the Children's Home—and as their guests all the children belonging to the married attendants and tradesmen on the asylum estate are invited also. The gathering takes place in a large hall filled with tastefully decorated tables; at one end a platform, on which the Woodilee orchestra and choir are assembled; at the other, a serving buffet, giving forth an aroma of newly infused tea from shining urns.

Around the tables are seated about 130 children, with their guardians, their happy faces beaming with anticipation of the treat in store for them, and an appetite ready to do justice to the array of good things provided.

As the choir chant their beautiful grace, sung at every meal, all rise to their feet, remaining until the closing words permit them to attend to more material things.

The medical staff, the matron, and her assistants help the little ones to tea, and more especially those sugared cakes so dear to the heart of youth.

But even over those luxuries the visitors are impatient, for is not Santa Claus in person to present gifts from a "real" Christmas-tree in the concert hall?

Christmas-tree at Woodilee Asylum.

As they wend their way up the thickly carpeted stair the excitement increases in volume. Those who had seen last

**WOODILEE ASYLUM, GLASGOW**—*continued.*

year's treat desire again to taste, while those without retrospective anticipation are even more eager, for it is theirs to have a first view of Father Christmas.

Climbing up the staircase they pass into an immense hall, illuminated to its furthest corners by the brilliancy of the gaily decorated tree. The electric lamps of varied colours cast their light in scintillating rays over rosettes, tassels, coloured balls and ornaments, producing innumerable hues, which, blending with the green of the spruce, make a happy background for the wealth of toys.

A buzz of suppressed excitement echoes round as they seat themselves into a ring, and, the choir under able leadership, sing "Good King Wenceslas."

Nearing the close of the carol the traditional figure of Father Christmas enters the room. His entry is the signal for loud applause, and as he stands, ruby visaged, beaming with enjoyment, you would almost imagine one of your childhood's ideals had appeared in the flesh.

Many willing hands dexterously cut off the presents, and hand them to Father Christmas, who, in a stentorian voice, announces the name of the recipient, and it is a pleasure to see the little ones coming forward to receive their gifts, some shyly and timidly, some boldly and respectfully, but all uttering a hearty "Thank you" as they accept their several souvenirs. No one is forgotten, and it would be hard to find a gathering wherein joy more abounds.

As the tree gets nearly stripped, Santa Claus retires amidst a storm of applause, and as the hall lights up the tree fades into darkness amidst audible sighs from the youngsters.

The orchestra plays an overture while the seats are arranged to enable the youngsters to see what wonders the cinematograph has to reveal, and while the ear is delighted with the harmony of the music, none the less is the eye with the appointments of the hall.

The polished floor, the pannelled roof, the walls with flattened Corinthian pillars, decorated in brown and gold, reflect the myriads of lights from the chandeliers, and vie with the proscenium in producing the brightest spectacle.

The cinematograph exhibition is invariably chosen entirely with a view to pleasing the children, and a series of amusing episodes run riot on the screen, amidst the laughter of the children and their elders.

After the entertainment a service of beef-tea and buns winds up the evening, which lasts from four till seven P.M. Then the little visitors troop home to dream over their glimpse of fairyland, lighting their way over the estate with hand lamps, which gives a pretty and quaint appearance to the grounds as the miniature illuminated procession of happy excited children passes out of sight.

### Derby Borough Asylum.

FOR a fortnight before Christmas patients and staff at Derby Borough Asylum are busily employed in decorating the various parts of the building, and as the result of their labours the customary somewhat bare and barrack-like wards are transformed into veritable gardens with artificial flowers, evergreens, and bunting. Appropriate mottoes are freely displayed everywhere, and the whole place bears a thoroughly bright and cheerful Christmas appearance.

FEMALE WARD, DERBY BOROUGH ASYLUM.

The patients themselves seem affected by the prevailing atmosphere of festivity and join heartily with the staff in the decorative work, and, in fact, almost everyone about the institution seems to have determined to forget everything but Christmas, and to be united in the endeavour to make it a really happy time.

A concert and dance for the patients on Christmas Eve may be said to start the celebrations, but the real beginning is made with the service on Christmas morning conducted by the chaplain in the chapel, which, in common with the rest of the building, is most tastefully decorated.

Pipes and tobacco are then given out to the men and appropriate gifts to the women, and the time spent quietly till 12.30, when the traditional fare of roast beef and plum pudding is partaken of in the dining-hall by all the patients who can possibly be allowed to join. The feature of the dinner is the plum-pudding course, all the puddings being brought in and placed on the tables blazing.

In the afternoon a sacred concert is held, in which various members of the staff take part.

Fruit and sweets are distributed during the day, and help to mark it as a very special occasion, and from beginning to end no effort is spared to make every patient feel that Christmas is indeed a time of rejoicing, and that in an asylum, as much as, if not more so than, in other places, goodwill towards all men is the sentiment that animates everyone.

The festivities do not end with Christmas Day itself. During the following week the holiday spirit still prevails, and entertainments are arranged for both the male and the female patients.

For the men there is a smoking concert, got up by the medical superintendent, with the assistance of friends from the town. Cigars, cigarettes, and tobacco are handed round, refreshments provided, and altogether a thoroughly enjoyable evening is spent. On another evening a Christmas tree and an entertainment takes place for the female side, organised by the ladies interested in the Brabazon Employment Scheme, a branch of which is connected with the asylum.

Almost all the women gather in the hall and receive well-selected gifts from the tree, and then after tea settle themselves to enjoy an excellent concert varied with dancing. The participation of the nurses and attendants in the festivities of the season is ensured by the household ball, to which they all invite their friends, and, free from all thought of duty and the cares of patients, enjoy their off time as much as is possible.

### Staffordshire Asylum, Cheddleton.

For weeks before Christmas the artistically disposed are busy preparing mottoes, shields, paper flowers, and other devices. There are many pottery designers among the patients, so that the standard is high, including water-colour sketches, cartoons, excerpts from the works of famous authors or musicians, and mirror frost work effects in magnesium sulphate. The week before Christmas is occupied in decorating the wards, entrance corridors, and dining-hall. About six tons of evergreens are kindly presented by members of the Visiting Committee.

On Christmas morning there is an early celebration for the staff. At ten choral communion service is held in chapel, with full orchestral accompaniment.

First staff mess is at twelve, the fare being goose, roast beef, plum pudding, and dessert. Patients' dinner is at one—roast beef, two vegetables, plum pudding, apples and oranges. Second staff mess is at 2. In the afternoon the parole patients go out to visit their friends, many for a country walk. Visitors to the wards are numerous, various small concerts and socials being given, including mumming or carol singing by boys from Cheddleton Village. In the evening there is a fancy dress staff dance.

Boxing Day is observed as a general holiday. At 6 p.m. a patients' soirée is held, and each worker receives a present—pipes, pouches, ties, collars, or gloves for the men, fichus, ribbons, shoes, or lace petticoats for the women. The tea tables are arranged for eight or ten each, and men and women sit together. The fare is ham and potted meat, sandwiches, buns, mince pies, cake, bread and butter, and jam. After tea comes a miscellaneous concert and a dance. Breaking-up time is about 10.30.

Flash-light of Recreation Hall during Patients' Ball, Cheddleton Asylum.

The staff supper is the most favoured of all the entertainments by the married workmen and artisans, although most of the indoor staff also attend. The fare is quantitative rather than qualitative—turkey, fowls, veal and ham and beef steak pie, apple tart, jellies, beer, claret cup, and lemonade. After supper games, cards, and dancing are kept up until 1.30.

The patients' concert is generally a huge success, on account of the amount of latent talent disclosed. Small parties or individual members of the staff supply songs, dances, stump speeches, sketches, etc., and no one knows what anyone else is going to do. A strong feature is parodying the weak points of former entertainments, nor is the administration sacred from topical hits.

STAFFORDSHIRE ASYLUM, CHEDDLETON—*continued.*

On New Year's Eve comes the patients' fancy-dress ball, and many are occupied for weeks over their disguises. It is wonderful to see how sullen paranoiacs or chronic melancholics unbend and cheer up for the nonce under the influence of the unwonted surroundings. Dancing goes with great spirit, for this is the only occasion in the year when the full orchestra of forty plays dance music. At midnight "Auld lang syne" is sung.

Twelfth night is generally devoted to theatrical performances, and so the Christmas season ends.

### West Malling Asylum, Kent.

Perhaps the most interesting feature of our present Christmas festivities has been the revival of the good old custom of carol singing in a very effective form.

The Church at West Malling Mental Asylum.

A splendidly trained band of carol singers, 45 in number, has been organised in Malling for the purpose not only of giving pleasure and perpetuating Christmas associations among the residents, but for the more practical purpose of raising subscriptions to give 250 poor children a good Christmas dinner and an evening entertainment.

This choral band honoured the asylum by volunteering to sing carols at different points all around the house, and they met with a gratifying reception by all the residents in the course of their visit.

The grand volume of well-harmonised vocal melody, with instrumental accompaniment, in such carols as the "Gloria in Excelsis" and "Nowell," heard in the murky darkness of a winter evening, will not soon be forgotten by those who were privileged to listen to it.

Christmas Day itself is principally marked by the appearance at dinner of the usual turkeys, geese, roast beef, plum pudding, mince pies, etc., and crackers also.

Christmas Day proper is followed by mirthful days and still more mirthful evenings. Golf, hockey, football, are to be seen by day, and the dance, drama, duologue, and music take turns in giving pleasure after the lights are lit and outdoor pleasures are impossible. Last year there was an amusing revival of Morton's famous "Box and Cox," the excuse for the revival being the histrionic ambition of a youthful lady of eleven years who undertook to play the part of Mrs. Bouncer herself, and played it well too, bringing down the house with the famous bolster "containing a handful and a half of feathers at each end and nothing at all in the middle." Merriment waxed still louder. She indignantly brought to the front the maligned bolster, with the scathing remark, "The idea of Mr. Cox presuming to complain of such a bolster as this!"

### York Retreat.

A stranger with the ordinary fanciful and vague notion of the interior of a mental asylum would probably be puzzled to say where he was if suddenly by magic set down in the quiet gallery of the York Retreat.

Everything seems bright and cheerful enough. Light and warmth are everywhere, and the Christmas evergreen abounds.

Here and there, sitting on a lounge or standing at a door of one of the drawing-rooms, may be seen a few ladies with something of an air of expectation in their attitudes. There is an atmosphere of "something about to happen." As a matter of fact, Christmas is beginning; it is now Christmas Eve. Only the hurrying past of a uniformed nurse gives a hint as to the nature of the institution.

And now expectation becomes realisation. A sound of singing is heard in the distance. Very soon it takes recognisable form. A procession of nurses, maids, and a patient or two, followed by tenors and basses drawn from the other side of the house, is approaching, and the familiar tune of an old carol breaks forth, clear and strong as the double line turns the corner and enters the gallery.

As the procession moves along there may be many whose memory takes them back to happier times when, in their own homes, they listened to the same familiar strains. But the kindly attentions of the nurses, and the amount of spirit thrown into the singing, are calculated to very quickly dissipate any feeling of this kind.

Let us follow the example of a few patients, and such nurses as can be spared from duty, and join the procession in the rear. Belle Vue, the detached villa from which our procession started, has already been visited, and a few carols have been sung in the drawing-room. Then a visit is paid to the Cottage Hospital, where one of the nurses is isolated after a spell of scarlet fever. After singing two carols for her especial benefit (and, incidentally, to the edification of the neighbouring terrace), the choir makes its way to the Retreat, and now follows in the wake of Father Christmas, who, assisted by a fairy Prince and Princess, goes a little beforehand, graciously dispensing gifts from the mysterious depths of a large basket. These gifts are from members of the Society of Friends in the neighbourhood of Westminster, who are following an example set in previous years by friends in York, Leeds, Manchester, Birmingham, Darlington, Croydon, etc. No patient is forgotten, and in some years it is possible also to include every member of the staff.

The choir lifts up its voice and sings lustily as it passes through the house from the ladies' side to the gentlemen's galleries upstairs, and back along the centre, to include more galleries on the female side. Then, after a visit to the kitchen and a march to the Gentlemen's Lodge, the tour of the main buildings is complete.

We have not done yet. Again we march forth into the cold night air. Even if the thermometer stand very near freezing, the East Villa must not be neglected, and so once more we "carol gaily" for the benefit of the ladies in residence there.

The old saying, "no song, no supper," next receives an illustration, and a very merry party, hoarse, but happy with a sense of duty done, has yet energy left for a vigorous attack on mince pies and coffee, before separating with seasonable wishes.

But the energy of our carol-singers is apparently inexhaustible, for "in the morning, oh so early," of Christmas Day itself a few enthusiastic nurses again uplift their voices in various parts of the grounds; and, if the festival fall on a Sunday, at the Evening Service a selection is once more given by the choir.

Thus begins Christmas at the Retreat.

But no account of the Christmas festivities would be complete without mention of some of the annual entertainments that follow Christmas Day.

The speciality last year was the tea-party on Boxing Day, for those ladies whose pecularities are too marked to allow of their joining in the more general parties. Although these guests include the patients who are the most demented and wanting in self-control, they all enjoyed the party and behaved well. It was necessary to have a strong staff of nurses in the room, and the doors had to be kept locked. Under these circumstances dancing and games were carried on with great gusto. First of all the curtain went up, revealing on the stage the tea-things and the nurses ready to minister to the enjoyment of their charges. The nurses then sang a carol, and the tea proceeded. The whole function lasted two and a half hours—from 3 to 5.30—and, with the exception of some vocal exercise more or less uncalled for, everything was orderly and satisfactory.

A particular feature, of course, of Christmas Day is the dinner; and a pleasant party takes place in the evening at the Medical Superintendent's house, where tea and coffee are provided, and there is much merriment over the "games."

But the chief events, known at the Retreat as the Christmas parties, do not take place until after Christmas. These assume the shape of two dances and three dramatic performances. Every patient and member of the staff who can suitably do so is invited to "join the revels."

The dramatic performances last year consisted of a comedietta, "The Nettle" and our old friend "The Rivals"—not done in its entirety, and, if the truth must be told, adapted to suit the Retreat stage. To quote from a satirical Society newspaper, it was "Sheridan out-Sheridaned." In the intervals between the acts music was supplied by members of the staff and visitors.

Amongst the actors in both piecees old friends with new faces were recognised and welcomed, whilst a hearty reception was accorded to new talent. The success of former Christmas festivities makes up hope that as each fresh Christmas comes round it may bring as good cheer, bright singing, clever acting, and enthusiastic dancing as its predecessors have done.

### Christmas Books.

Each Christmas as it comes round seems to bring with it a finer selection of gift-books, both as to style, get-up, and the very high level of the reading matter.

### Wells Gardner, Darton and Co.

All boys and girls will thoroughly enjoy "Happy-Go-Luckies," by M. H. Cornwall Legh (3s. 6d.); it deals with a family of delightful children, who, on account of straitened means, all go and live together on an old schooner, moored in a creek at the mouth of a river. They christen the boat "The Happy," and happy-go-lucky the family certainly are, from the parents downwards. The story is full of interest and excitement to the end. "How Things Went Wrong," by Raymond Jacberns (2s. 6d.) is an excellent story of life at a girls' school. Jean Bradley is a little terror at first, but after a series of scrapes and adventures she finds her level, and settles down into a charming child. The book has some good illustrations by Kate Street. "Three Little Conspirators," by Helen Beaumont (1s. 6d.), is a pretty tale dealing with the doings of a pair of mischievous twins and their little Indian cousin who comes to live with them. "Violet's Lent," "Tom and Bear," "The Cornishmen," by Evelyn Hunt (6d. each). These are nice little books, with a very good tone about them, though the moral is not too obvious. They are prettily illustrated by Agnes A. Hilton. "Prudent Paulina," by G. M. George and G. M. C. Fry (1s. 6d.) is an amusing little story told in verse; the illustrations are very comical. The story of Bayard can never be retold too often, and "Without Fear and Without Reproach," by F. T. H. Darton (1s.), is a most interesting account of the life of this heroic figure. Country children who do not possess the privileges of little Londoners will be delighted to read "The Zoo, Past and Present," by A. T. Elwes and the Rev. Theodore Wood (2s. 6d.), and the children of London themselves will hail with joy a book which gives them so many interesting details about their old friends. The illustrations are excellent. "The Queen of Shindy Flat," by Bessie Marchant (2s.), is an uncommonly exciting story for boys. The scene is laid in Venezuela, a country where startling events are the order of the day. Most of the characters get involved in a revolution on a small scale, and we read of adventures with alligators, boa constrictors, and jaguars; whilst a fire and an invasion of locusts also find their places in the narrative. Prickly Witchett, the "Queen" of the title, is an original character, and renders invaluable services to the kind people who befriend her. The next tale, "Terraweena," by Russell Allanson (1s.), takes us into Australia, and relates the doings of four schoolboys during their holidays. We think that most English lads will envy these boys the adventures they met with, ending by being kidnapped by blackfellows. The book contains some capital descriptions of life in the "backblocks."

### Ward, Lock and Co.

"The Wonder Book, a Picture Annual for Boys and Girls" (3s. 6d.), is charmingly got up, with numerous illustrations that are most attractive. Louis Wain, Ethel Turner, and Cecil Alden are among the contributors to this handsome book. There are some very pretty fairy tales that are sure to give delight, and a great many good stories of boy and girl life, at school or otherwise. We have a distinctly original tale this year in "The Story of the Gravelys," by Marshall Saunders (3s. 6d.). It is described as a book for girls, but we are sure it cannot fail to please all, both young and old. The characters are delightfully natural and the whole tone of the book is very healthy and bracing. Ethel Turner has produced a most interesting collection of stories this year, entitled "A White Roof-Tree" (3s. 6d.), which is the first and longest of the tales. Here we read of an enterprising family of boys and girls, who become "dwellers in tents" in order to save house-rent. The story is very breezy and sure to please. The short tales that follow are grouped under the heading "Sketches in Drought Time," and are full of interest and excitement. "Andy," by Lucile Lovell (3s. 6d.), is a pretty story of a small American boy very much of the "Little Lord Fauntleroy" type. Andy is a fine little fellow, who wins his way, all un-

**CHRISTMAS BOOKS**—*continued.*

daunted by many rebuffs, to his stern uncle's heart. The tale is so naturally told and the interest so well kept up that it is sure to find many absorbed young readers. Of much sterner stuff is "A Maker of History," by E. Phillips Oppenheim (6s.), wherein Guy Poynton, an English boy, travelling on the Continent, all unwittingly makes history by becoming an unseen spectator of a most fateful meeting between the Czar and Kaiser, and secures a page of a signed treaty between the two monarchs. Thereafter all the secret police of Europe are on his track, some as friends and some as foes. Which are the most unscrupulous we leave the many readers of this exciting book to discover; the distinction is a fine one. The Dogger Bank incident is made use of with telling effect, and the whole story is one of breathless interest. "She" has returned again, and we read of her wondrous doings in "Ayesha," by H. Rider Haggard (6s.). Leo Vincey and Harold Holly, our old and tried friends, go through even more thrilling and blood-curdling adventures than of yore in their search after "She," who is this time located in Central Asia, at the summit of a "fire-mountain." The hunting of the death hounds is almost as grisly an episode as the never-to-be-forgotten "Hot-Potting" in the former book. The illustrations by Maurice Greiffenhagen are wonderfully effective, and add much to this weird and enthralling narrative.

(*To be concluded.*)

### Central Midwives Board.

A meeting of the Central Midwives Board was held on Thursday, November 30, to consider the removal of the names of certain midwives from the roll. There were present: Dr. Champneys (chairman), Dr. Dakin, Mr. Fordham, Miss R. Paget, Miss Wilson, and Mr. Parker Young.

The first case heard was that of Mary Ann Coit, who, being in attendance on a patient on August 16, 1905, was charged with disobeying Rule E 2 and Rule Œ 11. Also Rule E 17 and E 19 (b). The circumstances, as related at the inquest, were briefly these: The child was born at 9.10 p.m. on August 16. The midwife was present, and stayed until about midnight, but did not wash the patient, and did not return until 4.30 p.m. on the following day. On the 18th she came again, and found the patient feverish, but did not take the temperature, not knowing how to use a thermometer, and in the evening a doctor was sent for, and subsequently a second came. The woman died on the 25th, and her death was attributed to puerperal septicæmia, caused by the neglect of the midwife to wash her.

Dr. Howlett said he could not speak except by hearsay until the 19th, when he took charge. He found the patient suffering from fever. He had been told that she had not been washed until nineteen hours after her confinement, and this he had, at the inquest, stated to be, in his opinion, the cause of septicæmia. He had conducted a post-mortem examination; he found nothing within the uterus, the woman was quite healthy, and he could not attribute her death to anything but want of cleanliness. The midwife had been suspended for a month by the Local Supervising Authority.

A letter was read from Mrs. Coit, who said she had given up practice, and had been ill ever since the case; she did not think she had done anything wrong, and she had come at ten o'clock the following day, not at four o'clock as stated.

The Board decided to remove her name from the roll, and in giving the verdict the Chairman said he wished it to be clearly understood that it was not the Board who were responsible for admitting women on the roll who were totally ignorant of the use of thermometers and such appliances. They were obliged by the Act to admit such people, even though they were of course not really fit for the work.

Annie Broomhead, certified midwife, was the next defendant, against whom the following charges were made: that whilst attending a patient on July 8, 1905, she disobeyed Rule E 6 and Rule E 17 (b). The woman herself appeared, and Dr. Boobyer, Medical Officer of Health, prosecuted on behalf of the city of Nottingham, the Local Supervising Authority.

Dr. Boobyer said that Mrs. Broomhead reported stillbirth on July 10, and on the 13th she was brought by the lady visitor to his office to explain the matter. She said that she knew the rules, but did not heed them, and had not sent for a doctor because she could tell when it was necessary to do so and when not. Dr. Boobyer said she was very contumacious, that her hands were very dirty, and she did not seem altogether temperate when she came to his office; he would not, however, say she was under the influence of alcohol.

Mrs. Broomhead said she attended the woman on the 8th about 1 p.m.; that the waters had then broken; that she went again in the afternoon, and in the evening about 10, and again at midnight, staying until six on Sunday morning. She denied that there was any presentation up to this time. She came again about five o'clock, and found the head and hand presenting. In cross-examination Mrs. Broomhead said she never left her after she found the hand presenting; that she had suggested to the woman that she had better have a doctor, but the woman was poor. She did not know the meaning of the words antiseptics and clinical thermometer.

The Board returned a verdict to the effect that the charges were not proven. In addressing Mrs. Broomhead, the Chairman said that she must consider herself extremely lucky, and that it was only necessary to convict her of not using antiseptics and a thermometer at her next case for her name to be removed, which would certainly be done without compunction; that she was not safe to practise.

The Board then considered the following: That Elizabeth Fetch, whilst in attendance on a patient on August 23, 1905, disobeyed Rules E 11, 2, 17, and 19 (a). Mrs. Fetch was represented by Dr. Hill, of Sheffield. The case against Mrs. Fetch was that she had attended a patient on the 23rd, the day of her confinement, and subsequently on the 24th, 25th, 26th, 27th, and had then ceased to attend until sent for by the husband on the 31st. Mrs. Fetch found the patient cold and thirsty, but did not think it necessary to send for a doctor. On the 9th of September, however, Dr. Burn was sent for, and said he should have been called earlier; on the 13th the woman died of puerperal septicæmia.

Dr. Hill, for the defence, said that Mrs. Fetch attended the patient until she was apparently quite well; on September 2 the woman was out of doors. He admitted the third and fourth charges, but said that the woman had with her scissors, vaseline, and permanganate of potash.

The Board censured Mrs. Fetch with respect to the third and fourth charges, acquitting her on the first and second, and advising her to become a monthly nurse. The Chairman pointed out that if Mrs. Fetch did not procure adequate instruction another course would have to be taken with regard to her ignorance if charges were again brought up against her.

Dr. Hill, in thanking the Board, said that he had merely defended the woman from a sense of justice, as she was a decent woman and much superior to the other two midwives in the parish, which contained 8,000 inhabitants, and was one of the poorest. He had often advised the woman to give up the work on account of her age (65) and her want of knowledge. He did not approve of such women being on the roll at all, and employed a certificated midwife himself.

### Presentation to Miss Thorold.

The great appreciation of all who have been connected with Miss Thorold during the thirty-five years in which she has so ably filled the post of lady superintendent of the Middlesex Hospital was amply attested on Thursday, November 30, when she was presented with an exceedingly handsome service of plate. The long list of subscribers included many of the governors, nurses, students, past and present sisters, and the nursing staff.

The lecture theatre in which the presentation took place was crowded, the assembly being largely composed of sisters and nurses. Miss Thorold's appearance on the platform, accompanied by her successor, Miss Vernet, was greeted with much enthusiasm. Lord Cheylesmore, in a few well-chosen words, testified to the regret felt by all at the loss of Miss Thorold after so many years of strenuous work, and introduced Lord Sandhurst, who made the presentation. It took the form of a handsome service of plate engraved with Miss Thorold's initials and contained in a large oak chest. The service included two dozen table and dessert spoons and forks, one dozen teaspoons, one dozen fish knives and forks, six candlesticks, one large silver bowl, one 14-inch waiter, besides many other articles. The gift of the nurses past and present consisted of a $3\frac{1}{2}$-pint kettle, stand, and lamp, coffee-pot, sugar-basin, large teatray, and a brooch. There was also a framed address, upon which the names of the subscribers (nearly 500 in number) were given. The address ran as follows : " Presented, with a service of plate, to Miss G. M. Thorold, upon her retirement, by numerous governors, members of the honorary medical and surgical staff and past and present students of the Middlesex Hospital, as an expression of their personal regard and appreciation of her administration of the nursing department of the hospital during a period of thirty-five years as lady superintendent. November 30, 1905."

Lord Sandhurst, addressing Miss Thorold, said he felt sure that she would appreciate even more than the value of the gifts the long and comprehensive list of subscribers' names. He had had only one serious quarrel with Miss Thorold, and that was when he discovered her at 5 A.M. sitting before her desk hard at work. He remonstrated with her severely, and obtained, as a great concession, the promise that she would in future retire to rest not later than 2.30 A.M. When Miss Thorold first took office in 1871 the old order was passing, and a new one was being established. A new system takes immense energy and strength, and she had proved herself fully equal to the demand upon her, admirably performing all her onerous duties. In bidding her farewell, Lord Sandhurst expressed the hope that Miss Thorold would enjoy her well-earned rest.

When Miss Thorold rose to speak she received a most enthusiastic ovation, and it was some moments before she could make herself heard. Although she was evidently deeply moved, she managed to express in a charming little speech her great appreciation of the beautiful gifts, and warmly testified her gratitude to all, concluding with the simple and convincing words, "From my heart I thank you."

Mr. Andrew Clark remarked that it was unnecessary to extol Miss Thorold's virtues. He could only endorse all that had already been said. He proposed a vote of thanks to Lord Sandhurst, which was carried with acclamation.

Lord Sandhurst, in reply, ventured to hope that his next visit to the Middlesex Hospital would not be the occasion of the resignation of one of the staff.

Miss Thorold has a retiring pension of £200 a year.

### "Queen's" Superintendents in Conference.

BY ONE WHO WAS THERE.

For the last five years an annual Conference of Queen's superintendents in the Northern Counties has been held. Three times the superintendents of the South have met in London in the same way, but this year it was decided by the united committees that it would be interesting as well as helpful to have a conference consisting of all superintendents of Queen's District Nurses' Homes in England, and to also invite those able to come from Scotland and Ireland.

The Council of the Institute showed their approval of the scheme by generously providing luncheon and tea for the guests, and visitors from the North received hospitality from their London *confrères*.

From 12.30 to 1 o'clock Miss Amy Hughes, the General Superintendent of the Queen Victoria Jubilee Institute, received the guests at the Hotel Windsor, Victoria Street. There were many pleasant meetings, old friends trained together in hospital, or as "Queen's Pros" in district work, some of them after many years, and making acquaintance with new friends. At one o'clock luncheon was served at small tables, and, as soon as they were cleared away, seats were arranged. By 2.5 Miss Rosaline Paget took the chair, and the business began.

The first paper was read by Miss Walker, of Bolton, who introduced the subject, "How Inspection can be made of the Most Use." Miss Darragh, one of the inspectors, replied, and the discussion soon became general and animated—frank suggestions being given and taken with all friendliness.

The second subject was "How to Utilise Outside Help," which was opened by Miss Mills, of Liverpool, and followed by Miss Curtis, of Hammersmith, dealing with the advantage to the district nurse of working with the friendly visitor to the poor—the Charity Organisation Society and other institutions.

The third subject was "The Relation of 'Queen's' Nurses to the Local Supervising Authorities under the Midwives Act," introduced by Miss Morgan, of Cardiff, and followed by Miss Andrew, of Gateshead. Miss Paget was called upon to reply to some of the difficulties met with in dealing with this subject.

"Registration" was dealt with by Miss Amy Hughes, who pointed out the importance of "Queen's" nurses coming to the right conclusion as to their position with regard to this burning question.

Votes of thanks to Miss Hughes, the Council of the Institute, the hostesses in London, Miss Paget for presiding, and Miss Maule for making all the arrangements, were unanimously passed. Then Miss Paget called upon Miss Blower, as the Queen's nurse whose name had been longest on the roll to make the presentation to Miss Peter.

Miss Blower, in a few well-chosen words, spoke of the love which all Queen's nurses bear to Miss Peter, and of the honour they felt it to have been associated with her, and asked her acceptance of an art-silver inlaid box, containing a cheque for £110 and a parchment with the names of the contributors to the remembrance.

Miss Peter replied with much feeling, repeating Queen Alexandra's message to her nurses, and thanking all who had worked with and for her during the seventeen years she had been connected with the Institute.

Tea followed, when there was a further opportunity of friendly chat, and at 6.30 the beautiful room placed at the disposal of the Queen's superintendents was empty once more.

### Nursing the Victims of the Charing Cross Disaster.

When the news that a terrible disaster had happened at Charing Cross Railway Station reached Charing Cross Hospital on Tuesday afternoon, immediately after the event, word was given to Miss Heather-Bigg, the matron, to provide as many nurses as she could to help in the Casualty Department. The matron accordingly sent the sisters and as many nurses as could be spared from the wards to the out-patient department. Here the large central waiting hall had been converted into a temporary ward into which all bad cases were taken, the sisters and nursing staff, of course, all working under the direction of the surgeons and house surgeons. Every available dresser and all the residents exerted themselves with the utmost energy during the evening in the interests of the sufferers. Ultimately, about thirty-five patients were able to leave, eight patients being subsequently transferred to the wards. The advantage of the close proximity of a great hospital with a fully equipped staff to a railway terminus was certainly never better illustrated.

### Unclaimed Medals for South African Nurses.

We are officially informed that medals for the following nursing sisters and maidservants who served in South Africa, and whose addresses are unknown, await issue at the War Office :—

*Nursing Sisters.*—F. H. Anderson, B. Agnes, E. Broadhurst, M. M. Buchinger, M. Baker, G. Baker, E. Baker, D. Betton, M. Beck, C. Bissett, B. Bamber, B. Bromilow, J. Bouchier, M. A. M. Bridges, M. E. Brindley, A. Buxton, M. Brice, A. Campbell (A.N.S.R., now Mrs. Doherty), W. Cochin, M. R. Clowes, M. Corcoran, C. Cowper, T. Davis, C. D. Dick, E. Dupont, E. E. Deane, A. M. Donegan, C. E. Davis, D. Davidson, H. F. Ferns (A.N.S.R.), C. Fishwick, Mrs. Field, E. Fathers, F. Galwey, M. Greenfield, M. Galloway, M. Huxton, L. L. Healy, T. Handleion, F. A. R. Hardman, M. de H. Harrison, K. C. Hodges, G. Hamman, N. Harker, M. Hoffe, G. Hannah, H. Jones, F. A. James, E. Karghin, M. Knox, F. E. Le Roux, M. Lloyd-Harris, L. Louch, E. Lindsay, C. A. McCloghery, D. Maritz, L. M. Moreton, E. Matthews, E. Masters, M. Martell, C. Macleay, K. M. A. Morrissy, C. Maclean, M. H. Nettleton, E. Nettle, L. R. Owen-Davis, M. Ormond, G. Ormond, F. Pollard, H. C. Parker, J. Potgeiter, L. Pain, A. Peddie, N. Read, S. Rodgers, L. Roper, E. Robertson, H. Ritchie, H. M. E. Ross, S. Stanford, Z. L. Smyth, L. Stewart, D. Schoch, O. Smith, I. Tuyt, F. Truter, N. S. Turner, K. Taylor, M. H. E. Uys, J. P. Van Benge, J. Williams, F. Wilson, Mrs. Wainwright, B. Wright, K. Wigley, J. Wilson, L. M. Wright, S. Wood, A. Webber, F. Whitehead, C. L. Watson, M. Yeoman. *Maidservants.*—L. Botha, J. Bovenizer, F. Colgan, E. Clarke, M. Davids, A. Goodall, K. Kelly, Mrs. G. Kelly, M. Martell, C. Robinson, M. A. Skillman, Mrs. E. Ward.

Applications for these medals should be made to the Secretary, War Office, 68 Victoria Street, London, S.W.

### Westminster Hospital Bazaar.

On Thursday last week, in aid of the Westminster Hospital, a sale of articles left over from the Historical Bazaar was held at 43 Bryanston Square, the residence of Colonel and Mrs. Gordon Watson. Lady Mary Howard, one of the Vice-Presidents of the Westminster Hospital Ladies' Association, opened the sale, and soon after business became brisk, and there was a fair number of visitors during the afternoon. The stall-holders were seven in number, and included the matron and some sisters and nurses from the hospital. An excellent tea and a string band added to the enjoyment of the afternoon, and Miss Lilla McCarthy gave some charming recitations. The wet weather kept a good many visitors away. About £62 was realised. There are still some goods left over, and it has been arranged for a sale of these to be held in the Board-room of the hospital on Thursday next at 4.30 p.m., to which there will be no charge for admission. All goods will be sold at half-price, and an auction will be held at the end. During the afternoon Mr. Calverley Bewicke will recite "The Queen's Gift," by the kind permission of the proprietors of *Punch*, and the Rev. F. H. Fare will give a lecture on Westminster Abbey, with lantern views.

### A Notable Memorial Service.

On Monday last a memorial service was held at St. Mary's Hospital, Paddington, for Mr. Joseph Huggins, hospital porter, who had faithfully worked in the institution for twenty-one years, and who passed away on the previous Wednesday after five days' illness. The service was at 2 p.m., and, though the very busiest time of the day, each ward was able to send a representative. The choir stalls were well filled. The choir, led by the home sister and Sister Boynton, sang "On the Resurrection Morning." Several members of the visiting staff were present, and as many of the resident medical officers and students as could be spared. The coffin was covered with wreaths sent by members of the staff, the matron, sisters, and nurses, and two from the porters, six of whom carried the remains of their old friend and colleague to the grave.

### Association for Promoting the Training of Midwives.

As a result of the Council Meeting of the Association for Promoting the Training and Supply of Midwives, held on October 27, an informal meeting of the Association took place last week at 2 Cromwell Houses, South Kensington, when Mrs. Samuel Bruce laid before the members the details of the scheme, which she had proposed at the Council meeting referred to, for enlarging, on systematic lines, the circle of subscribers to the funds of the Association. Mrs. Bruce's scheme is, shortly, that London should be divided into districts, and that ladies should undertake to interest their friends and acquaintances in certain localities in the objects and work of the Association, and thus, by annual subscriptions, to ensure a definite income whereby the training of midwives, and other work undertaken by the Association, may be enlarged and carried on without the anxiety as to funds which must exist when an organisation is supported largely by donations.

### Royal Boscombe and West Hants Hospital.

On Thursday last week the "Irene" Ward for Children was declared open by Mrs. Kenneth Balfour. Those who know the difficulty of nursing the children in the wards with acute cases of men and women will understand the extreme need of the undertaking decided upon by the committee. An electric light has been put between each bed, and can be moved any way required. At the back of the ward are isolation quarters, with lavatories and bathroom. The whole is built on arches, and, when necessary, can be hosed everywhere. The matron and nurses are proud of their hospital, which has a pleasant air of homeliness and comfort.

### A New Book for Surgical Nurses.

The Scientific Press, Limited, are issuing this week a new work entitled "Surgical Instruments and Appliances" by Harold Burrows, M.B. This book should prove of the utmost assistance to those upon whom the duty of making arrangements for a surgical operation may fall. The list given represents as nearly as possible what may be regarded as the average requirements, and the nurse will not go far wrong in following them to the letter. The fact, too, that the book is very fully illustrated renders it of great help in understanding the various appliances.

### Meeting of the Nurses' Union.

The Medical Missionary Sale of Work of the Nurses' Union was held on Thursday last at 5 Cambridge Gate, Regent's Park. Miss Dashwood was "At Home" to members of the Union and their friends from 3 to 8. The object of the sale was to raise the sum of £10 for the support of the native nurse in Lucknow and £10 for the Nurses' Union bed in the hospital at Nablus. After tea in the dining room, Mrs. Richardson, the only English lady nursing the soldiers wounded in the late Japanese war, gave a vivid and interesting account of her work. There was then a pause so as to give time for making purchases, and at 6.30 p.m. a Russian lady, who has also been a missionary in China, took the chair, and greatly interested the nurses with a few details about her own experience.

### "The Hospital" Convalescent Fund.

The Hon. Secretary begs to acknowledge, with thanks, the receipt of 2s. 6d. from Nurse Barnes Groom, and 2s. 6d. from the Travel Correspondent of The Hospital.

### Everybody's Opinion.

### CHRISTMAS NOT ALWAYS A JOY.

"Another Member of the Royal National Pension Fund" writes : You have invited correspondence on the subject of Christmas presents in hospitals, and I write to say that in this hospital we have now but one general collection at Christmas. This is for a present to our matron, and we should none of us wish to discontinue it. We receive many kindnesses from her, and it is a pleasure, not a tax, to subscribe to a present once a year, as a small token of our affection and regard. The subject has been a good deal discussed since the letters and article appeared in The Hospital, and the nurses all agree that it would take away a great deal of the pleasure of Christmas if they could not continue their usual custom in this respect. Each one gives what she wishes and can afford, and no one except the collector knows who gives and what each gives, and we do it because we like and wish to, not because we feel we must.

### NURSES AND PARTY POLITICS.

"Marion Chadwick, Chairwoman of the Women's Liberal Unionist Association, Kensington Branch," writes from 19 Phillimore Gardens : Having to-day seen the paragraph in your issue of November 25 with reference to the meeting held on November 15 for the discussion of the problem of State Registration of Nurses, will you permit me to make one correction as to fact. The meeting was held under the auspices of the Kensington Branch of the Women's Liberal Unionist Association, and was got up with a view of giving our members real information on both sides of this important question. You will surely agree with our Association in thinking that women ought to educate themselves in all questions that may become the subject of Legislation, and that when discussions are held in such a noncontentious way as I think we may claim for our meetings, it would be a great mistake if nurses or other people who have the facts at first hand were to refuse to assist in thus helping to form public opinion."

### PRIVATE CONFERENCES ON PUBLIC QUESTIONS.

Miss Lilian A. Maule, Secretary of the Metropolitan and Southern Counties Association of Queen's Superintendents, writes : Your comment on the recent Conference of "Queen's" superintendents in your last issue is both unfair and misleading, your argument being based upon grounds that are entirely inaccurate. Allow me to point out that the "organisations" meeting to confer—*i.e.* the two Associations of Queen's Superintendents in the northern and southern counties—are in no sense "dependent for existence upon public money." They are private Associations, dependent solely upon the private subscriptions of the members, and they have no official connection whatever with the Queen's Institute, though the Council cordially recognise their usefulness, and in the present instance have kindly contributed towards the expenses of the Conference by private donations. The questions discussed are concerned with the intimate details of the work of a district superintendent, and anyone who understands the matter will at once realise that the whole object of these Conferences, which are held to enable superintendents to consider between themselves these details, would be lost were the Press admitted. By the courtesy of the Committees I send you a report of the Conference, and I may add that had you written to me asking for such particulars as I could give you in advance, I should have been happy to comply.

[Even if the Associations are private organisations, supported by private contributions, it cannot be affirmed that Miss Peter's testimonial, to which 500 Queen's nurses subscribed, was a private function. With regard to the official report of the Conference—which reached us on Tuesday evening—we had already been provided with an account from an independent source.—Ed. The Hospital.]

### Appointments.

British Home for Incurables, Streatham, London.—Miss Langman has been appointed staff nurse. She was trained at St. George's Hospital, London, where she afterwards became staff nurse and sister.

Coventry and Warwickshire Hospital.—Miss Marian E. Cresswell has been appointed night sister. She was trained at the North Staffordshire Infirmary and Eye Hospital, Stoke-upon-Trent, where she was afterwards staff nurse. She has since been sister at Oldham Infirmary and temporary sister at Liverpool Children's Infirmary. She has also done private nursing.

Glasgow Lock Hospital.—Miss Alice Frisby has been appointed charge nurse. She was trained at Ecclesall Infirmary, Sheffield, where she has since been charge nurse.

Hampstead General Hospital.—Miss Hickson, Miss O'Dowd, and Miss Carter have been appointed sisters; and Miss Foucar and Miss Drake staff nurses. Miss Hickson was trained at the Poplar and London Hospitals. Miss O'Dowd was trained at St. Bartholomew's Hospital, London. Miss Carter was trained at Guy's Hospital, London. Miss Foucar was trained at the Temperance Hospital, London. Miss Drake was trained at West Ham Hospital.

Harrow-on-the-Hill Infectious Diseases Hospital.—Miss Emma Musker has been appointed nurse matron. She was trained at the Hope Hospital, Pendleton, Manchester, and she has since been staff nurse at the City Hospital, Grafton Street, Liverpool, staff nurse at the Hospital for Women and Children, Leeds, charge nurse at Stockton Fever Hospital, sister at the Hope Hospital, Pendleton, and nurse matron of the Birkdale Isolation Hospital.

Miners' Hospital, Skelton-in-Cleveland.—Miss Clara Baldwin has been appointed nurse-matron. She was trained at the North Riding Infirmary, Middlesbrough, where she has since been sister successively of Children's Ward, Male Medical Ward, and Male Surgical and Ophthalmic Wards.

Palmer Memorial Hospital, Jarrow-on-Tyne.—Miss M. Preston has been appointed staff nurse. She was trained at the North Riding Infirmary, Middlesbrough-on-Tees, and has since been staff nurse at Royal Infirmary, Derby.

Shoreditch Infirmary, London.—Miss Emily Duncan has been appointed superintendent night nurse. She was trained at St. George's Hospital, London, and has since been sister at Lewisham Infirmary.

Wisbech Workhouse Infirmary.—Miss Annie Louisa Clements has been appointed superintendent nurse. She was trained at Liverpool Poor-law Infirmary, and has since been charge nurse at Bradford Union Infirmary, charge nurse and night superintendent at Sunderland Poor-law Infirmary, and charge nurse at Norwich Poor-law Infirmary. She holds the certificate of the Central Midwives Board.

### Christmas Novelties.

(By Our Shopping Correspondent.)

### THE SWAN-WHITE APRON.

The swan-white apron is made of a waterproof material. It is in appearance almost exactly like the nice white aprons worn by nurses, with bib and shoulder straps. When soiled it can be cleaned without emersion in water, and very easily. It is a great improvement on the ugly nursery apron of mackintosh, and is moderate in price. The address of the manufacturers is The Red Lion Manufacturing Co., 71 High Holborn, W.C.

### ATORA BEEF SUET.

As a fat for frying Atora can be strongly recommended. It is also very useful in the making of cakes and flaky pastry. For the last purpose a liberal supply should be used. It is sold in solid blocks, or shredded for the convenience of the purchaser.

### PLUM PUDDINGS AND MINCEMEAT.

The plum-puddings and mincemeat which Messrs. Lazenby, of Trinity Street, S.E., send out ready prepared for Christmas use are excellent. They are to be procured at most grocers, but if there is any difficulty this firm is prepared to forward them direct. Where time and trouble must be saved this portion of the Christmas fare can with advantage be secured from Messrs. Lazenby. Both the puddings and the mincemeat are of good quality and delicious flavour.

### A DELICIOUS CHOCOLATE.

Every year sees new improvements in the decorative designs of Christmas presents, and not least among the pretty novelties displayed in the shop windows this year are the boxes in which Messrs. Fry have put up their delicious sweets. Of most artistic design are these *bonbonières*, some of which are cased in velvet of pastel shades with a bas-relief of classical design in the centre. The delicate flavour and wholesome quality of Fry's chocolate are widely recognised, and, altogether, these elegant boxes should prove a welcome item on our Christmas present lists this year.

### EXQUISITE PERFUMES.

Many Christmas shoppers recognise the fact that a bottle of really good perfume is a most acceptable present; and the scents made by Messrs. Nühlen, of Glockengarse, Cologne, which are obtainable almost everywhere where scent is sold, are very fragrant. The 4,711 Eau de Cologne is quite the best, and their Rhine Violets and other perfumes are almost without rival. Their sachets, soaps, and toilet requisites are all to be recommended.

### PRIVATE CHRISTMAS CARDS.

Some very pretty and effective greeting cards for the festive season can be obtained of the Scientific Press, Limited, 28 and 29 Southampton Street, Strand, W.C., at moderate prices ranging from 2s. upwards, which includes the printing of one's own wording, and envelopes.

### THE "LEWIS" FOUNTAIN PEN.

A very useful and acceptable Christmas present is the handsome fountain pen which Messrs. Lewis and Co., of 86 Mansion House Chambers, E.C., are offering at the moderate price of 2s. 6d., post free, complete with gold nib and filler.

### PENBERTHY'S PRESENTS.

That time-worn adage, "nothing new under the sun," must have originated long years ago in the superior masculine mind, but this is an age of progress and novelties fully appreciated at least by the frivolous mind of mortal woman. Those in search of Christmas gifts will find a large collection of pretty, dainty, and suitable articles to choose from at Mr. Penberthy's, 388 Oxford Street. In the fancy department there is a great variety of hand and wrist bags in crocodile, calf, morocco, and other kinds of leather. Some pretty coat of mail and silver purses, trinket and oxidised silver boxes from 1s. Silver photo and calendar frames, and some really uncommon hatpins with rolled gold heads in a simple but good design at 2s. There are also some charming little opera glasses enclosed in a morocco case, which occupy a minimum of space, at 21s. Apropos of operas—and even nurses occasionally indulge in such luxuries—one of the many pretty sights at Mr. Penberthy's is the assortment of fans, dainty, giddy, irresistible things varying in price from 2s. 11d. upwards, but some can be had costing 10s. 6d. made of white or black gauze, with a design after Watteau—steel and gold spangle relief and

One of Mr. Penberthy's Fans.

mounted on carved and gilt bone or ebony stick, and these are really charming. In another department may be seen numbers of the daintiest semi-evening and visiting blouses, crêpe-de-chine, mousseline-de-soie, velveteen, and delaine, frills, sequins, tucks, and lace yolks galore. Some of the smartest cost 18s. 11d. There are also some day blouses equally pretty made in soft fabrics and shades at 8s. 11d. Of gloves there is an endless variety, amongst many decidedly worth consideration are some of real reindeer at 3s. 11d., or, better still, at 5s. 6d.; they are splendid for wear and warmth—some of real gazelle in four colours which cost 2s. 11½d. Suède and kid for evening and day wear in all colours and prices. Those who want something really new should ask to see the "bracelet glove." It is stitched to represent a border of embroidery at the wrist, has two pearl buttons, and is extremely well finished and dainty, price 4s. 6d. But of the many delightful things to be seen the *pièce de résistance* comes under the heading of handkerchiefs. Tucked, frilled, scolloped, hand-embroidered and hem-stitched, made of real shamrock lawn, French lawn, cambric, pure linen, out of boxes they flutter like fleecy clouds, dainty enough for the most fastidious "faire ladye," and modest enough in price to suit the average purse, seeing that they vary from 10½d. to 5s. 6d. each. In the matter of lace handkerchiefs there is also a great variety in Honiton, Brussels, Irish guipure, etc., costing from 2s. 11½d. upwards. No one who pays a visit to Mr. Penberthy's can fail to find some suitable and pretty gifts for Christmas, and those who cannot get to town would do well to write for a catalogue.

### Notes and Queries.

**REGULATIONS.**

**The Editor is always willing to answer in this column, without any fee, all reasonable questions, as soon as possible.**

**But the following rules must be carefully observed.**

**1. Every communication must be accompanied by the name and address of the writer.**

**2. The question must always bear upon nursing, directly or indirectly.**

**If an answer is required by letter a fee of half-a-crown must be enclosed with the note containing the inquiry.**

*Rest Cure.*

(65) Would you inform me at your earliest convenience the name of the nearest hospital for the "Rest Cure?" Also, the name of the bone specialist in London?—*N. M.*

The rest cure could be carried out in any good nursing home or private hospital, but we cannot give names of private nursing establishments in this column, nor the names of specialists. Write to the Scientific Press, 28 Southampton Street, Strand, who will send you a list of private homes and specialists (price 6d.); and ask your local surgeon.

*London County Council.*

(66) Will you please let me know where to apply for the post of nurse in one of the London County Council Schools?—*V. B.*

Apply to the London County Council, Spring Gardens, S.W.

*Registration.*

(67) Will you kindly give me (1) your opinion and remarks about the Nurses' Registration Report, and (2) mention when and where to apply for registration.—*J. M.*

(1) We have no space here for criticism. (2) There is at present no State registration for nurses.

*Mental.*

(68) I should be glad to know of any home or convalescent home where they would take a young woman slightly afflicted in mind. She could only afford to pay very little.—*L. A. L.*

Perhaps the National Association for Promoting the Welfare of the Feeble-minded, 53 Victoria Street, S.W., might help you; or advertise, stating full particulars.

*New York or Washington.*

(69) Can you let me know the best way to procure the address of some private nursing homes in New York or Washington, and also would my Irish certificate from a large training school be recognised there?—*Nurse R.*

"The Hospital" circulates considerably in America, or you might advertise in the "American Journal of Nursing" or "The Trained Nurse," New York. Your Irish certificate, if for three years, would be recognised anywhere.

*Canada.*

(70) How can I obtain information about nursing in Canada? Can I join the Colonial Nursing Association stipulating to be sent to one particular colony?—*M. McM.*

"The Hospital" circulates in Canada, or you might advertise in "The Canadian Nurse," 133 East Bloor Street, Toronto. Write to the Colonial Nursing Association, Imperial Institute, W., and inquire.

*Royal British Nurses' Association.*

(71) Can you tell me if two years in a woman's hospital and the L.O.S. entitles me to become a member of the Royal British Nurses' Association?—*S. F. F.*

Members must have had three years' general training, but you can write to the Secretary, (10 Orchard Street, Oxford Street, W.), and state your case.

*Bandage-winder.*

(72) Where can I purchase a bandage-winder at a reasonable price such as is mentioned in "The Nurses' Clinic"?—*A. B.*

From the Holborn Surgical Instrument Company, 26 Thavies Inn, E.C.; or Messrs. W. H. Bailey and Son, 38 Oxford Street, W.

*West Australia.*

(73) Is there a good chance of a nurse who has general training and C.M.B. certificate being successful in going out to Western Australia to take up private nursing on her own account there?—*A. B.*

Not without influence or good introductions, and enough money in hand to keep you for 12 months if unemployed.

**Handbooks for Nurses.**

| | Post Free. |
|---|---|
| "How to Become a Nurse: How and Where to Train." | 2s. 4d. |
| "Nursing: its Theory and Practice." (Lewis.) ... ... | 8s. 6d. |
| "Nurses' Pronouncing Dictionary of Medical Terms." ... | 2s. 0d. |
| "Complete Handbook of Midwifery." (Watson.) ... | 6s. 4d. |
| "Preparation for Operation in Private Houses." ... | 0s. 6d. |

**Of all booksellers or of the Scientific Press, Limited, 28 & 29 Southampton Street, Strand, London, W.C.**

### For Reading to the Sick.

"OUR FATHERS."

Father of all, to Thee
With loving hearts we pray,
Through Him, in mercy given,
The Life, the Truth, the Way;
From Heav'n, Thy Throne, in mercy shed
Thy blessings on each bended head.

Father of all, to Thee
Our contrite hearts we raise,
Unstrung by sin and pain,
Long voiceless in Thy praise;
Breathe Thou the silent chords along,
Until they tremble into song.

*Julian.*

Does it not make all the difference in the world to us if we really believe that we have a heavenly Father? If we really know that the Almighty Governor of the whole world is not a tyrant, but a Father Who overlooks no soul that He has created, but attends to each and cares for each, and claims the love and service of each, impartially and individually; and Who, if for a while He hides His face from us, and disciplines us with sharp punishment, will certainly at the last give us the fullest measure of satisfaction of which we are capable—if we know this, I say, may not we feel the sun always shining in our hearts?

God's Fatherhood means, indeed, an infinite readiness to forgive. There is no limit to the number of times that God will accept our imperfect repentances, and encourage us to begin afresh. As long as we are capable of returning to choose God, God is assuredly willing to receive us. But all the same, we cannot fail to see that the Father may be finally driven to reject his sons. We can be sure that every man will have the fullest opportunity of knowing God and making his peace with Him in this life or beyond it.

By God's Fatherhood, we mean His Impartial Love. Back behind all in this world which seems so cruel, so unjust, so unequal, Christians believe there beats the heart of a Father, a heart of impartial love. This truth is constantly stated or implied in the New Testament. "God is love," and "With God is no respect of persons." "Ye call on the Father, Who, without respect of persons, judgeth according to every man's work."

God knows you and me, and acts upon you and me, as if there were none other in the world for Him to know and act upon. And this truth of God's individual love, Jesus Christ specially connects with His Name of Father.

*Bishop Gore.*

Father of all, to Thee
We breathe unutter'd fears,
Deep-hidden in our souls,
That have no voice but tears;
Take Thou our hand, and through the wild
Lead gently on each trustful child.

Father of all, may we
In praise our tongues employ,
When gladness fills the soul
With deep and hallow'd joy;
In storm and calm give us to see
The path of peace which leads to Thee.

*Julian.*

## Figures and Tables

**Figure f1:**
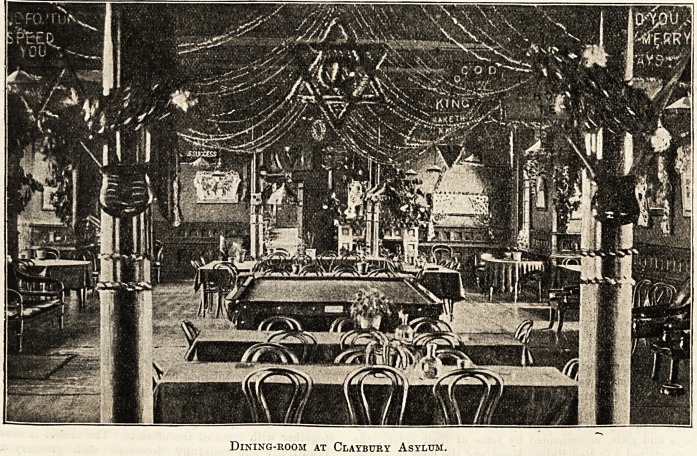


**Figure f2:**
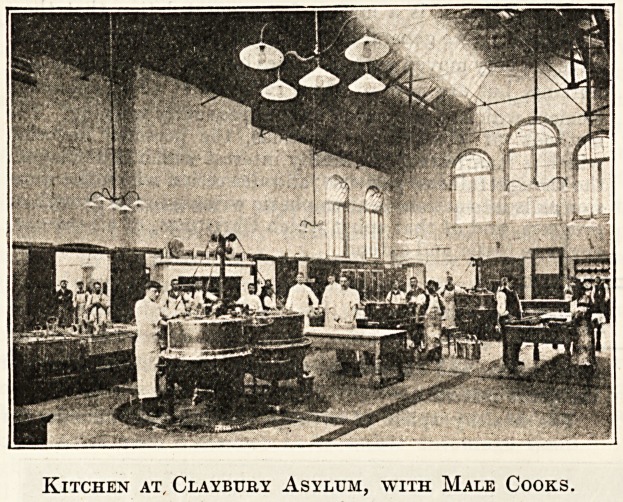


**Figure f3:**
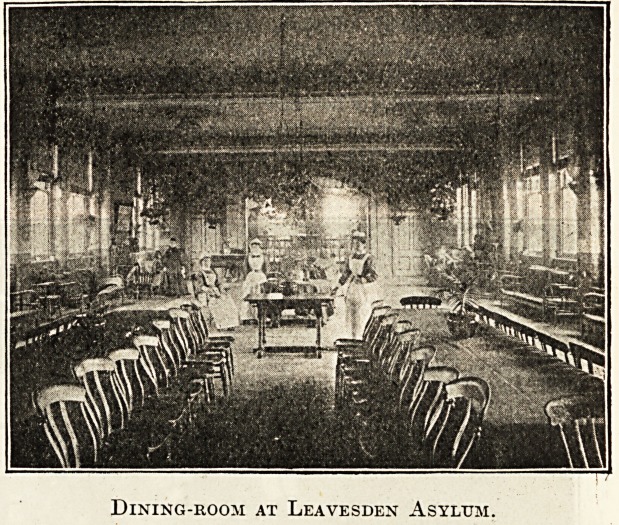


**Figure f4:**
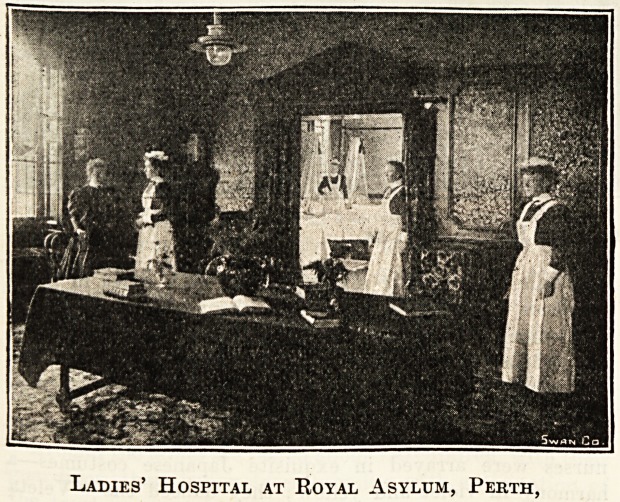


**Figure f5:**
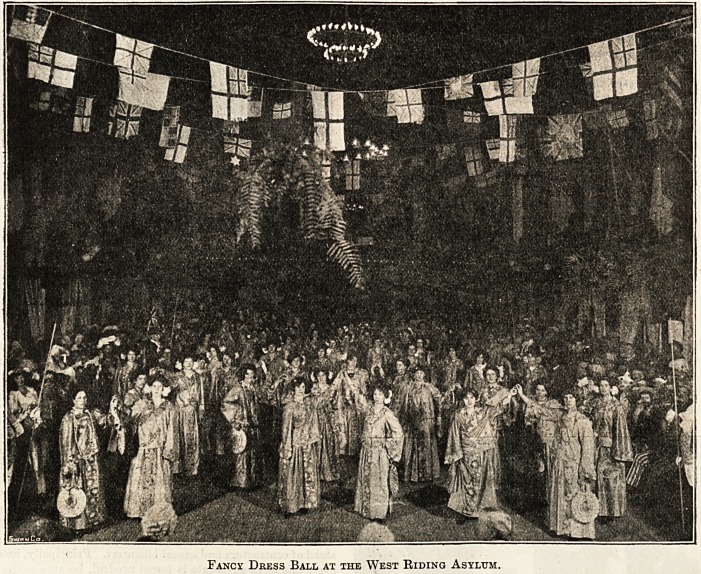


**Figure f6:**
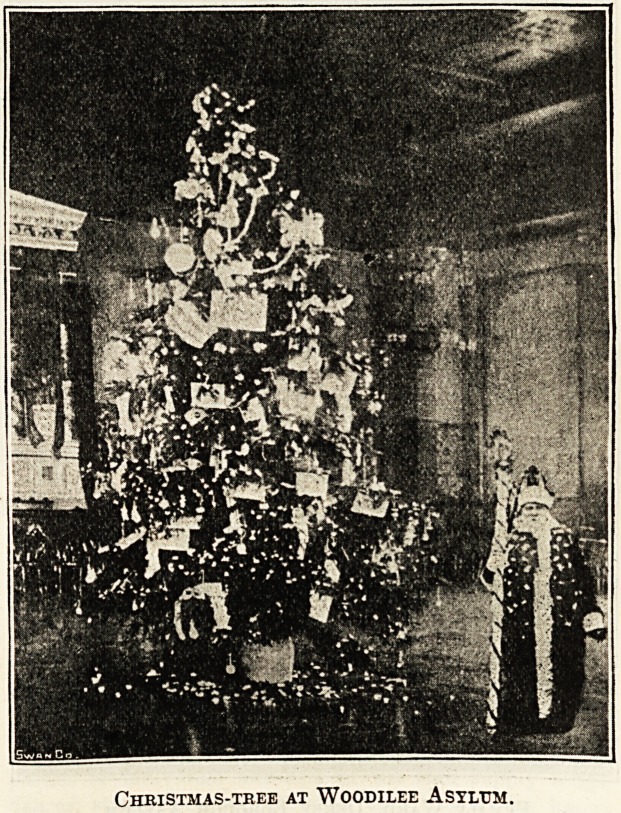


**Figure f7:**
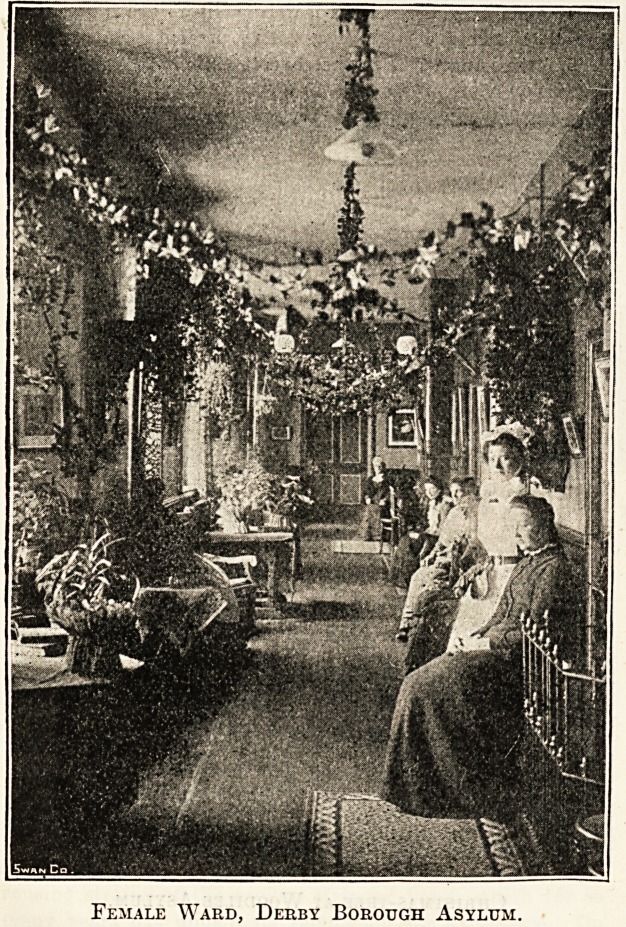


**Figure f8:**
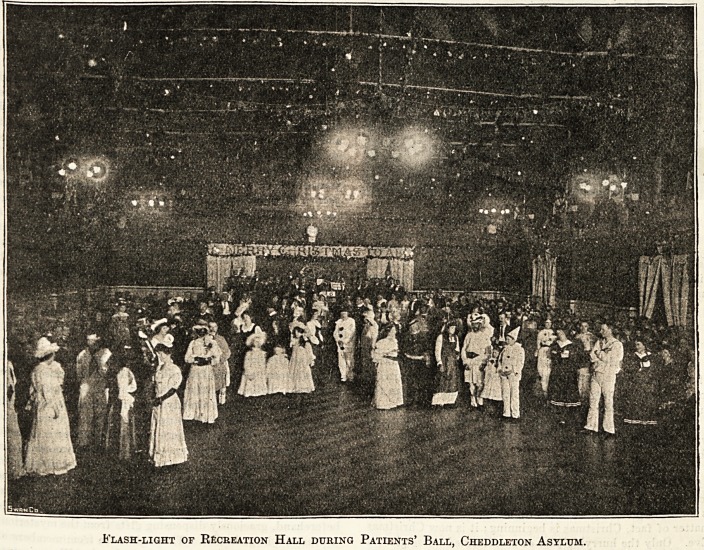


**Figure f9:**
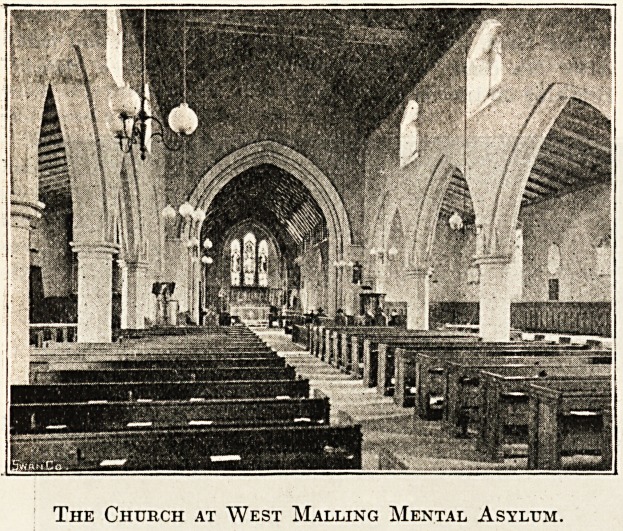


**Figure f10:**